# A novel and atypical NF-KB pro-inflammatory program regulated by a CamKII-proteasome axis is involved in the early activation of Muller glia by high glucose

**DOI:** 10.1186/s13578-022-00839-x

**Published:** 2022-07-16

**Authors:** Diego Sbardella, Grazia Raffaella Tundo, Alice Mecchia, Camilla Palumbo, Maria Grazia Atzori, Lauretta Levati, Alessandra Boccaccini, Anna Maria Caccuri, Paolo Cascio, Pedro Miguel Lacal, Grazia Graziani, Monica Varano, Massimiliano Coletta, Mariacristina Parravano

**Affiliations:** 1 IRCCS-Fondazione Bietti, Rome, Italy; 2 Department of Clinical Sciences and Translational Medicine, University of Rome Tor Vergata, Rome, Italy; 3 Department of Systems Medicine, University of Rome Tor Vergata, Rome, Italy; 4 IDI-IRCCS, Rome, Italy; 5 Center for TeleInfrastructure (CTIF), University of Rome Tor Vergata, Rome, Italy; 6 Department of Chemistry, University of Rome Tor Vergata, Rome, Italy; 7 Department of Veterinary Sciences, University of Turin, Turin, Italy

**Keywords:** Diabetic retinopathy, Muller glia, NF-kB pathway, Proteasome, CamKII

## Abstract

**Background:**

Diabetic retinopathy (DR) is a microvascular complication of diabetes with a heavy impact on the quality of life of subjects and with a dramatic burden for health and economic systems on a global scale. Although the pathogenesis of DR is largely unknown, several preclinical data have pointed out to a main role of Muller glia (MG), a cell type which spans across the retina layers providing nourishment and support for Retina Ganglion Cells (RGCs), in sensing hyper-glycemia and in acquiring a pro-inflammatory polarization in response to this insult.

**Results:**

By using a validated experimental model of DR in vitro, rMC1 cells challenged with high glucose, we uncovered the induction of an early (within minutes) and atypical Nuclear Factor-kB (NF-kB) signalling pathway regulated by a calcium-dependent calmodulin kinase II (CamKII)-proteasome axis. Phosphorylation of proteasome subunit Rpt6 (at Serine 120) by CamKII stimulated the accelerated turnover of IkBα (i.e., the natural inhibitor of p65-50 transcription factor), regardless of the phosphorylation at Serine 32 which labels canonical NF-kB signalling. This event allowed the p65-p50 heterodimer to migrate into the nucleus and to induce transcription of IL-8, Il-1β and MCP-1. Pharmacological inhibition of CamKII as well as proteasome inhibition stopped this pro-inflammatory program, whereas introduction of a Rpt6 phospho-dead mutant (Rpt6-S120A) stimulated a paradoxical effect on NF-kB probably through the activation of a compensatory mechanism which may involve phosphorylation of 20S α4 subunit.

**Conclusions:**

This study introduces a novel pathway of MG activation by high glucose and casts some light on the biological relevance of proteasome post-translational modifications in modulating pathways regulated through targeted proteolysis.

**Supplementary Information:**

The online version contains supplementary material available at 10.1186/s13578-022-00839-x.

## Key Points

- High glucose quickly induces an atypical NF-kB pro-inflammatory program.
- CamKII phosphorylation of Rpt6 subunit of the proteasome stimulates IkBα turnover and p65-p50 release.
- Inhibition of either CamkII or proteasome blocks this pathway.

**Supplementary Information:**

The online version contains supplementary material available at 10.1186/s13578-022-00839-x.

## Introduction

Diabetic retinopathy (DR) is a microvascular complication of diabetes which often leads to irreversible blindness, a clinical outcome which carries a heavy burden in terms of quality of life of affected subjects and economic-social costs on a global scale [1, 2].

DR pathogenesis evolves through two stages, referred to as non-proliferative (NPDR) and proliferative DR (PDR). NPDR is characterized by increased vascular permeability followed by haemodynamic alterations of choroidal and retinal vasculature, whereas the hallmark of PDR is neo-angiogenesis [1].

Muller glia (MG), a cell type which span across the neuroretina providing mechanical and nutritional support to the highly specialized retina neurons, has long been matter of studies aimed at figuring out DR pathogenesis, as it was reported to acquire a reactive phenotype in response to hyper-glycaemia in rat models of DR and in human retina isolated post-mortem [3–7].

Furthermore, prolonged (days) cultivation of MG cells in high glucose (≥ 25 mmol/L) medium was reported to induce the synthesis and secretion of: (a) pro-inflammatory cytokines (interleukin 1β [IL-1β], tumour necrosis factor α [TNFα], interleukin-8 [IL-8], monocyte chemoattractant protein-1 [MCP-1] and vascular endothelial growth factor (VEGF), a master regulator of neo-angiogenesis [8–10].

NF-kB is a master inflammatory pathway which regulates the transcription of these genes. In the canonical pathway, (for a comprehensive description of pathway refer to [11–16]), the NF-kB core transcription factor, composed by a p65 and p50 heterodimer, is kept inactive in the cell cytosol through the binding with the inhibitor of kinase B-α (IkBα), a family of natural inhibitors.

IkBα is a natively unfolded protein which has a very short half-life (few minutes) due to the presence of a PEST sequence in the C-terminus which promotes its ubiquitin-independent degradation by the proteasome [11]. When complexed with p65-p50, the PEST sequence is masked and IkBα gains stability and longer half-life (up to 8 h) [11, 14]. Release of p65-p50 then occurs through a signalling cascade induced by pro-inflammatory stimuli (e.g., TNFα and IL-1β or LPS) which involves the activation of inhibitor of kappa B kinase (IKK).

Once activated, IKK phosphorylates p65-p50-bound IkBα at Serine residues 32 and 36 promoting its ubiquitylation by the SCF^βTrCP^ E3-ligase and proteasome-mediated degradation. This renders the p65-p50 heterodimer free to enter the nucleus, where it binds to the promoter sequences of pro-inflammatory and anti-apoptotic genes, including IkBα, according to a negative feedback mechanism that has evolved to shut the transcriptional activity of this pathway off [11, 12].

Hence, regulation of NF-kB further requires a functional Ubiquitin Proteasome System (UPS), a major intracellular proteolytic pathway that stands at the crossroad of cell metabolism [17–20].

In the canonical structural configuration, the proteasome is made up by the 20S, a hollow barrel-shaped particle composed of four heptameric rings, two outer α rings and two inner β, which house the catalytic subunits (i.e., chymotrypsin-like, trypsin-like and caspase-like) complexed, at one or both α-ends, with the 19S [18, 21, 22]. The 19S is arranged into the base (Rpt(s) subunits) and the lid (Rpn(s) subunits) subassemblies which couple the affinity-recognition and the ATP-hydrolysis, to unfold and pull the poly-ubiquitylated proteins down into the 20S catalytic chamber [22–24].

Proteasome composition is constantly tuned to meet the metabolic need of the cells through binding with Regulatory Particles (RPs) others than the 19S which modulate its functional properties, as well as through post-synthetic modifications of subunits [25–29]. This last possibility is an emerging topic in proteasome biology, as phosphorylation, acetylation, glycosylation of selected subunits are being unveiled in pathophysiological conditions, though the functional significance is often unclear [30, 31].

Herein, we have carried out a characterization of the early dynamics of canonical NF-kB signaling and proteasome modulation by a hyperglycemic insult in rMC1 cells, a validated experimental model of Muller glia. High glucose (25 mmol/L) delivery was found to quickly induce an atypical nuclear translocation and transcriptional activation of p65-p50 through a Serine 32 phosphorylation-independent clearance of IkBα by the proteasome. This pathway seems activated through phosphorylation of the 19S Rpt6 subunit at Serine 120 by CamKII, an event that triggers the bulk degradation of intracellular poly-ubiquitylated substrates, likely shifting, through targeted proteolysis, the equilibrium of biological reactions regulated by short-lived proteins.

## Results

### Transcription of pro-inflammatory cytokines is induced early by high glucose concentrations in rMC1

**Fig. 1:**
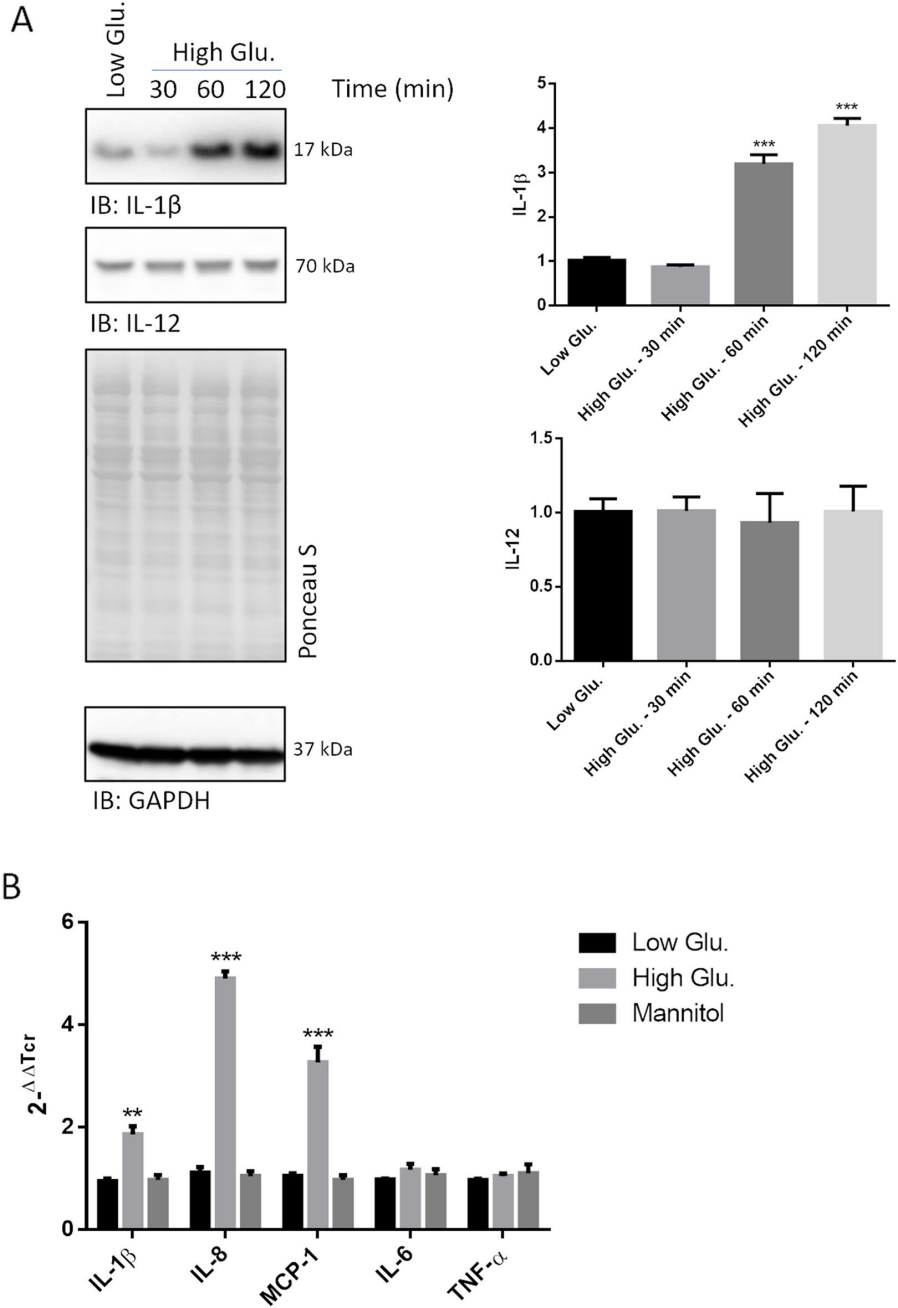
The transcription of pro-inflammatory cytokines in rMC1 cells is quickly induced by high glucose. **A** Whole cell extracts of rMC1 cells challenged with 25 mmol/L glucose and 5 mmol/L glucose or 20 mmol/L mannitol (a representative low glucose lane is reported in all figures showing immunoblots) for the indicated time-points were analysed by Wb (labelled as “IB” throughout all figures) and probed with antibodies raised against IL-1β and IL-12. Total proteins stained by Ponceau S were used as loading control. Histograms show the densitometric analysis of the immunoreactive bands; data are the mean ± SD of three independent experiments; a representative blot is shown. Statistics refer to the comparison between high and low glucose treated cells. One-way ANOVA followed by Tukey’s post-hoc test: ***p < 0.001. **B** RT-PCR analysis of a selection of pro-inflammatory cytokines in the same sample groups as in **A**. Transcript levels of high glucose treated cells were compared to low glucose and mannitol treated cells. β-actin was used as internal control. Data are presented as mean ± SD (n = 3); one-way ANOVA followed by Tukey’s post-hoc test (n = 3): **p < 0.01, ***p < 0.001

We preliminarily noticed that immunostaining of the 17 kDa IL-1β fragment (i.e., the biologically active species) was very robust in whole lysates of rMC1 challenged with 25 mmol/L glucose (referred to as high glucose throughout the text) for up to 2 h (Fig. 1A). The intensity of cytokine immunostaining was significantly lower in lysates of cells stimulated with 5 mmol/L glucose or mannitol, used as hyperosmolar control (referred to as “controls”). Conversely, immunostaining of IL-12 (Fig. 1A) and IL-10 (undetectable, *data not shown*), which is not regulated by NF-kB, was unaltered by treatments over the same time-interval.

To address whether the IL-1β increase reflected the transcriptional upregulation and not a post-synthetic mechanism (e.g., increased processing of pre-IL-1β or impaired secretion), a RT-PCR analysis of rMC1 challenged with 25 mmol/L glucose and controls for 40 min was undertaken. Compared to control cells, cells challenged with high glucose displayed a ~ twofold increase of IL-1β transcript (Fig. 1B). Thus, additional cytokines linked to NF-kB activation were enrolled in the study: IL-8 (~ fivefold increase) and MCP-1 (~ threefold increase) turned out to be induced to the greatest extent in high glucose challenged cells. Conversely, transcription of IL-6 and TNFα was not induced (Fig. 1B).

### An atypical NF-kB pathway is activated within minutes after rMC1 challenge with high glucose

**Fig. 2:**
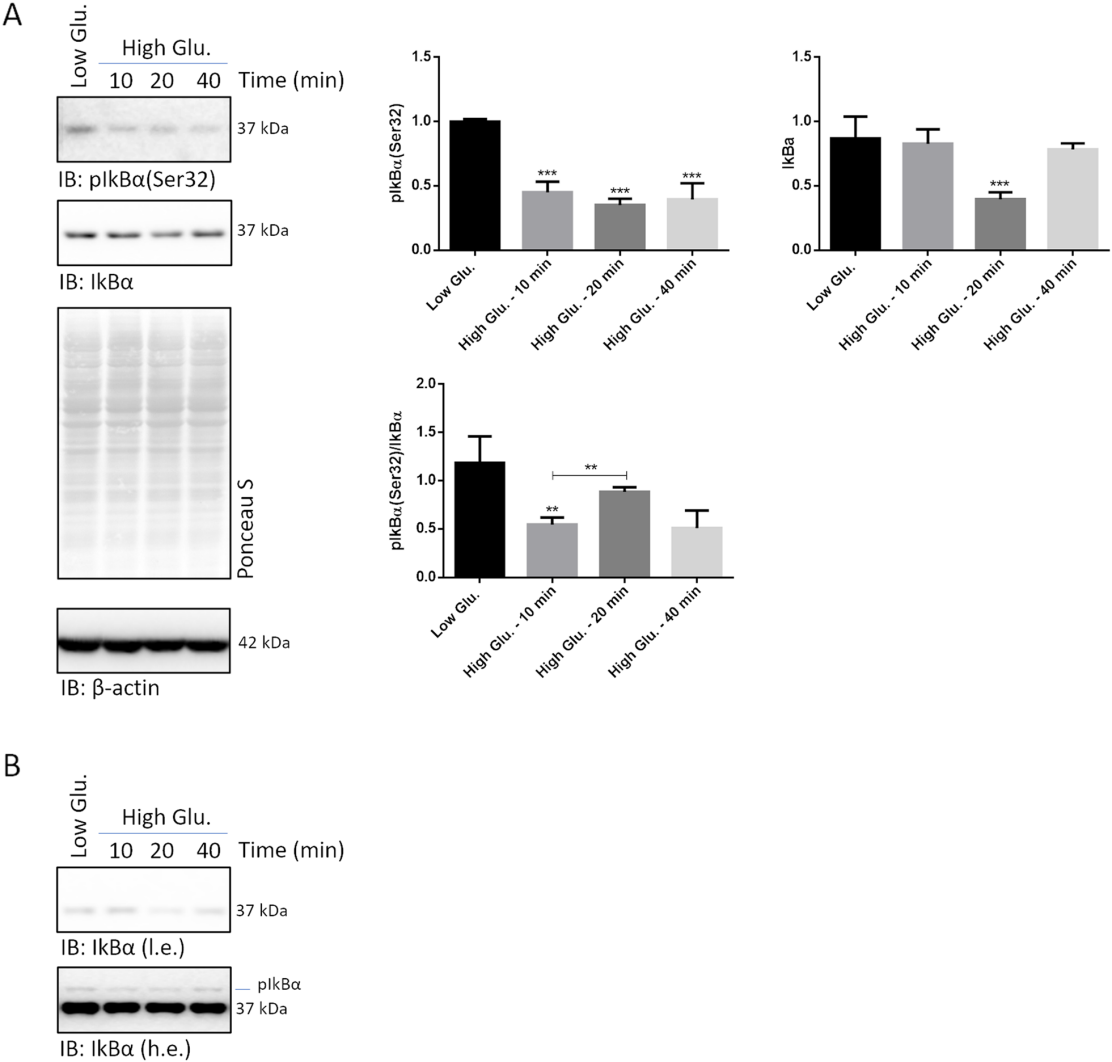

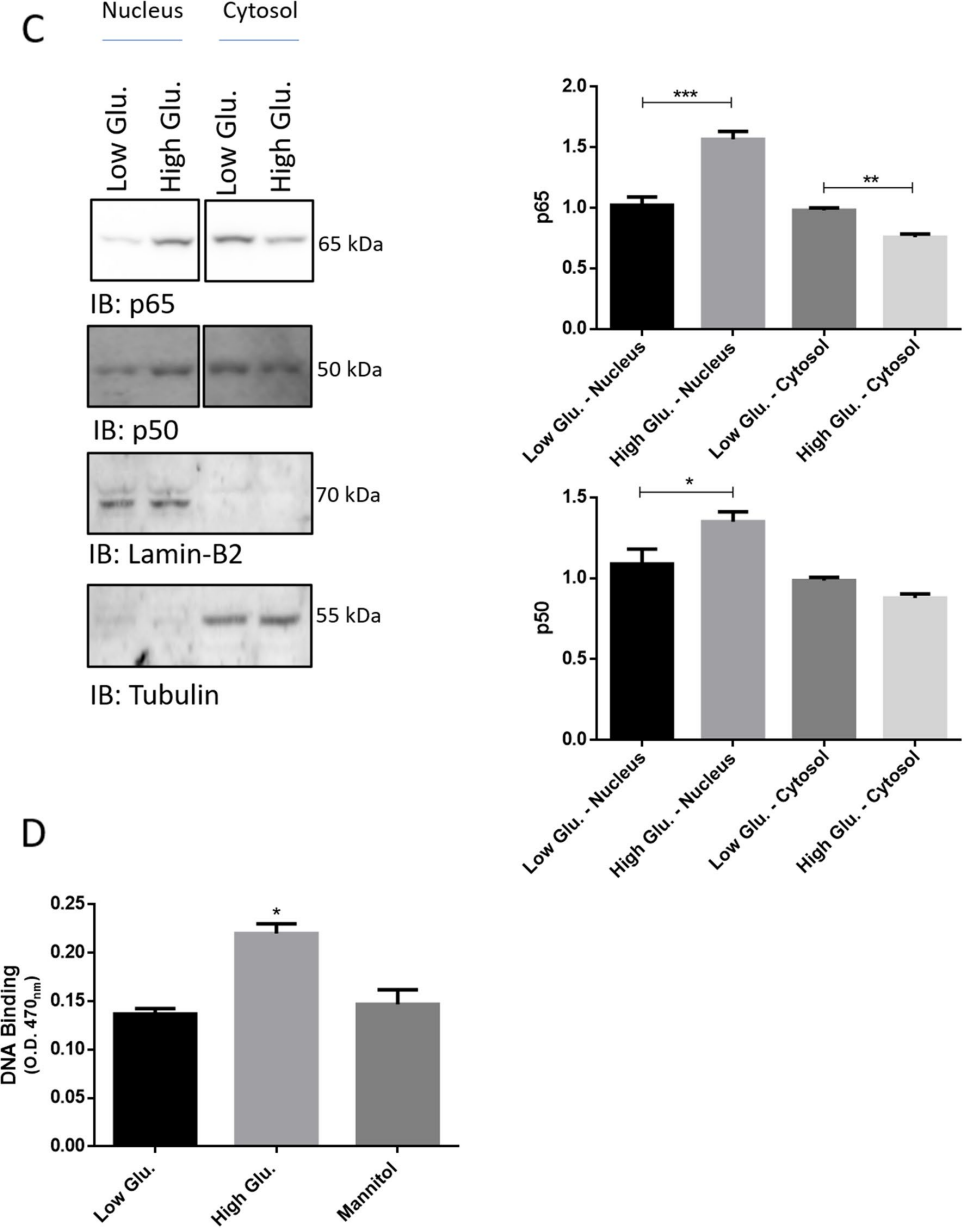
High glucose stimulation induces the nuclear translocation of p65-p50 regardless of IkBα phosphorylation. **A** Whole cell lysates were prepared from rMC1 cells challenged with 25 mmol/L glucose for 10, 20 and 40 min or from control cells and analysed by Wb. Filters were probed with antibodies anti-pIkBα(Ser32) and -IkBα. Total proteins stained by Ponceau S were used as loading control. Results of densitometric analysis are presented as Mean ± SD (n = 3); a representative blot is shown. Statistics were calculated for high glucose *vs* low glucose treated cells. One-way ANOVA followed by Tukey’s post-hoc test: **p < 0.01, ***p < 0.001. **B** Phos-tag analysis of the same samples as in **A**. The filter was probed with an anti-IkBα antibody. Low and high exposures of the filter are shown. **C** Nuclear and cytosolic extracts were isolated from rMC1 cells challenged with 25 mmol/L glucose and low glucose or mannitol for 30 min (preliminary settings showed that stimulation for 30 min provided results more robust than those observed after 20 min). Wb analyses of these fractions was performed by using antibodies anti-p65, -p50, -lamin-B2 and β-tubulin. Histograms represent the results of densitometric analysis of the immunoreactive bands. The intensity of p65 and p50 bands of high glucose treated cells was compared to that of low glucose treated cells separately for each compartment (e.g., nucleus or cytosol). **D** Nuclear extracts from rMC1 cells challenged with 5 mmol/L glucose, 25 mmol/L glucose or mannitol were analysed by Trans-AM immune-enzymatic assay and DNA binding by p65 was evaluated as O.D. read at 470 nm. Statistics were calculated for high glucose *vs* low glucose or mannitol treated cells in **C** and **D** and data are presented as Mean ± SD (n = 3); one-way ANOVA followed by Tukey’s post-hoc test: *p < 0.05, **p < 0.01, ***p < 0.001

To investigate whether high glucose may actually trigger an early activation of NF-kB, a time-course analysis of IkBα phosphorylation at Serine 32 [pIkBα(Ser32)] in whole cell lysates of rMC1 challenged for 10, 20 and 40 min with 25 mmol/L glucose and low glucose or mannitol was performed by Wb [11–13]. Compared to control cells, pIkBα(Ser32) immunostaining dropped after 10 min of stimulation with high-glucose, and was persistently decreased within the time-interval analysed (Fig. 2A). Immunostaining of unphosphorylated IkBα dropped as well, but, with respect to pIkBα(Ser32), it showed a 10 min delay: in fact, compared to control cells, the greatest decrease of total IkBα was documented 20 min after high glucose delivery (Fig. 2A; Additional file 1: Fig. S1). Thus, the pIkBα(Ser32)/IkBα ratio, usually very high during canonical NF-kB signalling, was markedly decreased at 10 min of high glucose challenge, but unaltered at 20 and 40 min (Fig. 2A).

To rule out the existence of unprecedented IkBα phospho-sites, a phos-Tag analysis was set up. Separation of lysates from control and high glucose treated cells allowed to detect a band pair compatible with unphosphorylated and phosphorylated IkBα (see *lower and upper* band in overexposed blot, Fig. 2B). However, high glucose did not cause any obvious increase of delayed-mobility species at any time-point investigated (Fig. 2B).

To verify whether pIkBα(Ser32) and IkBα drop was followed by nuclear translocation of p65-p50, the nuclear and cytosolic fractions of rMC1 stimulated with 25 mmol/L glucose and controls for 30 min were isolated and analysed by Wb. Immunostaining of p65 and p50 highlighted a significant increase of the two proteins in the nuclear fraction of high glucose *vs* control cells, mirrored by a drop in the corresponding cytosolic fraction (Fig. 2C). Lamin-D2 and β-tubulin immunostaining confirmed the identity of the two fractions and a negligible reciprocal contamination (Fig. 2C).

To further validate NF-kB activation the DNA binding activity of p65 was assayed in the nuclear extracts of cells challenged with high glucose and controls for 30 min by using a commercially available ELISA-immunoassay (Fig. 2D). The NF-kB DNA binding ability, measured by the absorbance at 470 nm (O.D.), was about twofold higher in high glucose than in control cells nuclear extracts (Fig. 2D).

### A second cycle of canonical NF-kB activation is observed at later time points in rMC1 exposed to high glucose

**Fig. 3:**
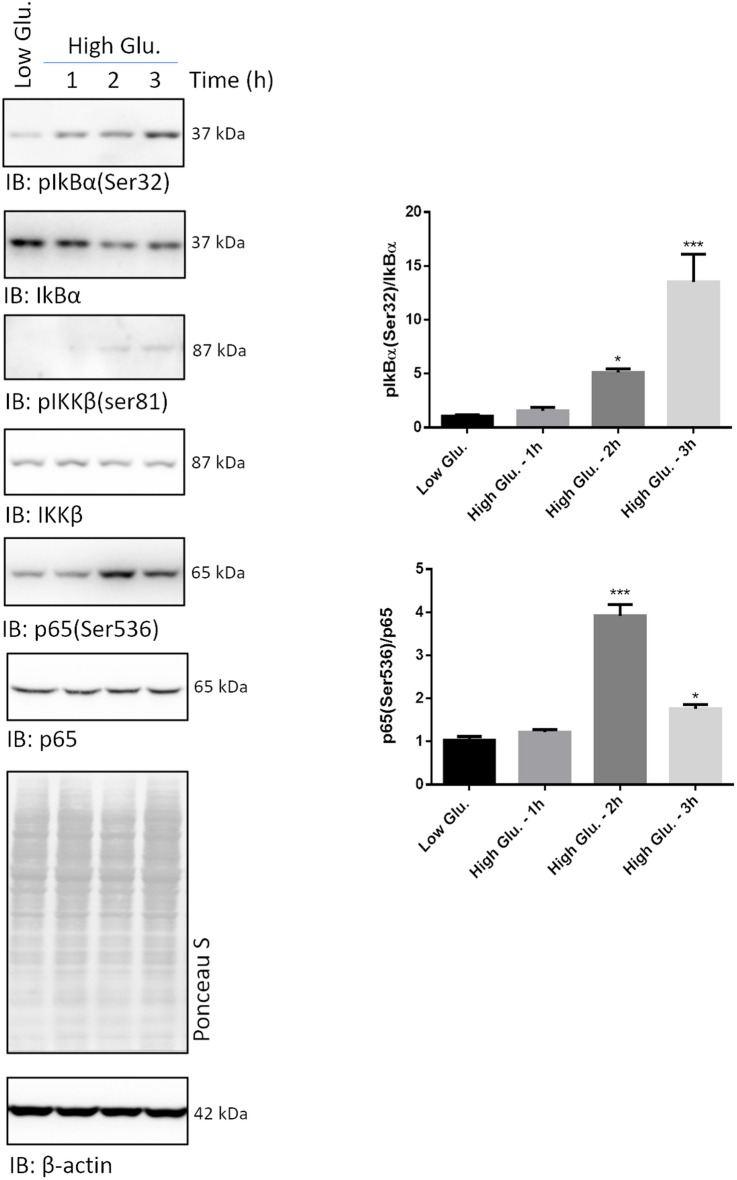
A second cycle of NF-kB activation by high glucose occurs through a typical signalling pathway. Whole cell lysates were prepared from rMC1 cells challenged with 25 mmol/L glucose and with low glucose or mannitol for 1, 2 and 3 h and analysed by Wb. Filters were probed with antibodies anti-pIkBα(Ser32), -IkBα, -pIKKβ(Ser80), -IKKβ, -p65(Ser536), and -p65. The pIkBα(Ser32)/IkBα and p65(Ser536)/p65 ratios are shown. Being pIKKβ undetectable in control cells, the unphosphorylated/phosphorylated ratio of this protein is not reported. Total proteins stained by Ponceau S were used as loading control. Histograms represent the results of densitometric analysis of the immunoreactive bands; data are the mean ± SD of three independent experiments; a representative blot is shown. Statistics were calculated for high glucose *vs* low glucose treated cells using a One-way ANOVA followed by Tukey’s post-hoc test: *p < 0.05; ***p < 0.001

Whole cell lysates were collected from rMC1 challenged with high glucose and low glucose or mannitol for 1, 2 and 3 h to figure out whether cytokines, once released, induced a canonical NF-kB signalling following a pro-inflammatory pathway. Compared to control cells, immunodetection of pIkBα(Ser32) progressively increased 2-3 h after high glucose delivery, whilst IkBα dropped significantly (Fig. 3). Hence, this time, the pIkBα(Ser32)/IkBα ratio was greater in high glucose than in control cells.

To confirm the occurrence of a canonical NF-kB signalling, filters were probed for unphosphorylated and phosphorylated IKKβ [at Serine 180, referred to as pIKKβ(Ser180)], and unphosphorylated and phosphorylated p65 (at Serine 536, referred to as p65(Ser536] (Fig. 3). After 2–3 h of stimulation, pIKKβ(Ser180) turned out to be detectable, in the absence of any variation of total IKKβ. Similarly, phosphorylation of p65(Ser536) significantly increased after 2–3 h of high glucose stimulation with respect to control cells. Also in this case, no modulation of the unphosphorylated p65 was observed (Fig. 3). Thus, the p65(Ser536)/p65 ratio was increased in high glucose treated (in particular at 2 h) compared to control cells.

### Proteasome bulk activity is stimulated by 25 mmol/L glucose in rMC1

**Fig. 4:**
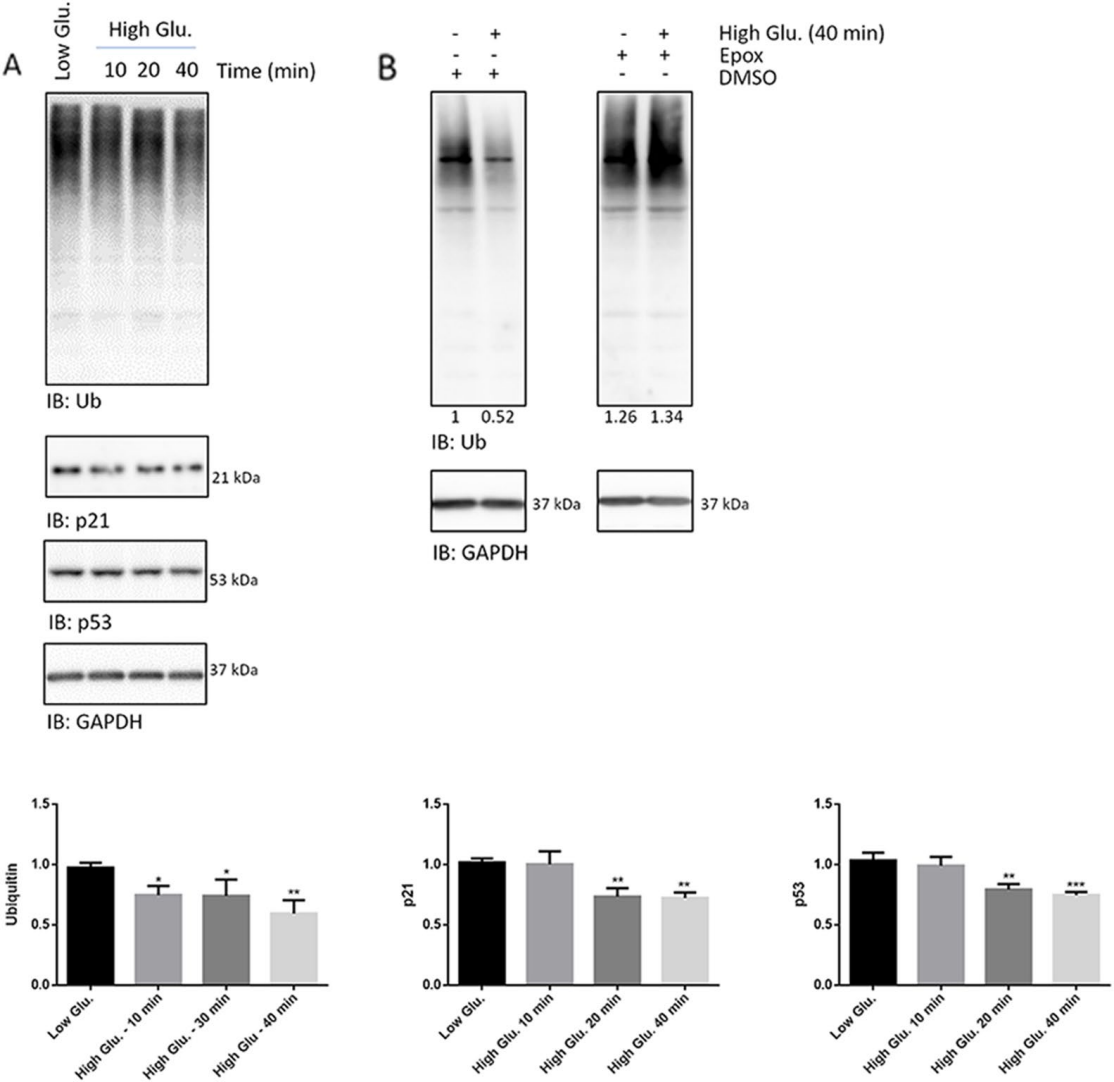

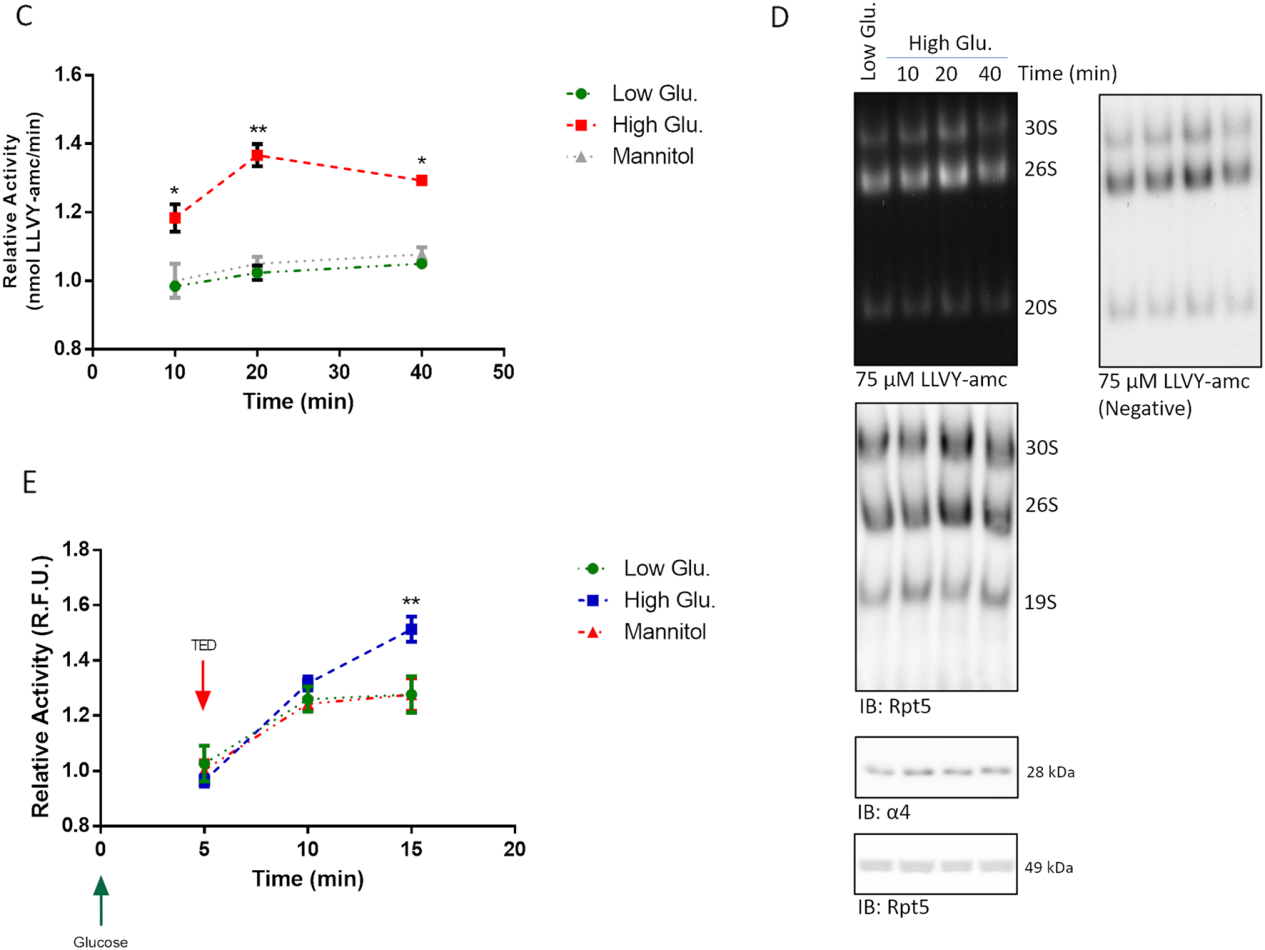
Proteasome bulk activity is quickly stimulated by high glucose delivery. **A** Whole cell extracts of rMC1 cells stimulated with 25 mmol/L glucose and with low glucose or mannitol for 10, 20 and 40 min were probed with antibodies anti-ubiquitin, -p21 and -p53; GAPDH was used as loading control by Wb analysis. Histograms show the densitometric analysis of the immunoreactive bands; data are the mean ± SD of three independent experiments; a representative blot is shown. Statistics were calculated for high glucose *vs* low glucose treated cells; one-way ANOVA followed by Tukey’s post-hoc test: *p < 0.05, **p < 0.01. **B** rMC1 cells were pre-treated with 500 nM epoxomicin or vehicle (DMSO) for 1 h. Thereafter, the compound was washed out before addition of high glucose or controls. After 40 min of incubation, whole cell lysates were prepared, analysed by Wb and probed with the anti-ubiquitin antibody. GAPDH was used as loading control; raw normalized values of band intensity are reported below the upper panel. **C** Crude cell extracts were isolated from rMC1 cells challenged with high glucose as indicated in **A** and bulk proteasome activity assayed on Suc-LLVY-amc, a fluorogenic peptide specific for the chymotrypsin-like activity. Values reported in the graph are the relative ratio of the slope calculated for high-glucose treated cells *vs* low-glucose or mannitol-treated cells at each time-point. Peptide cleavage was monitored until linearity was observed (~ 40 min). The time (x) scale refers to the timing after glucose stimulation; slopes have been determined by subtracting that obtained in the presence of 500 nM epoxomicin (added to the crude cell extract) for each experimental condition. Non-specific cleavage of the fluorogenic probe in the presence of epoxomicin was negligible. One-way ANOVA followed by Tukey’s post-hoc test (n = 3): *p < 0.05; **p < 0.01. **D** Crude cell extracts of cells treated with low glucose and high glucose were separated by native-gel electrophoresis. The activity of proteasome particles was visualized in-gel upon delivery of 75 µM Suc-LLVY-amc. The identity of capped species was determined by Wb analysis, probing the filters with an anti-Rpt5 antibody. An aliquot of these extracts was further analysed by denaturing and reducing Wb and probed with anti-α4, -Rpt5 and -Rpt6 antibodies; a representative experiment of three independent replicates is reported. **E** Living rMC1 cells were pre-stimulated with 500 nM epoxomicin or DMSO for 1 h. Thereafter, the compounds were washed out and the stimuli (i.e*.*, low glucose, high glucose and mannitol) delivered. After additional 5 min of incubation the TED fluorogenic peptide (17 µM) was added to the culture medium and fluorescence release monitored over 20 min of high glucose stimulation. Arrows indicate the precise timing of stimuli administration. Data are reported as relative comparison between Relative Fluorescence Unit (RFU) obtained at each time-point. In accordance with previous results, preliminary data indicated that TED half-life is quite short (around 30 min. and that non-specific cleavage of the peptide was almost null, since epoxomicin treated cells did not release appreciable fluorescence (data not shown) [32]. One-way ANOVA followed by Tukey’s post-hoc test (n = 3): **p < 0.01

Since IkBα is a prototypical proteasome substrate, to shed light on the NF-kB early activation, we verified whether high glucose delivery for 10–40 min actually induced a drop of ubiquitylated proteins by Wb. Compared to control cells, high glucose induced a robust decrease of ubiquitinylated proteins and a slight drop of p21 and p53, two reporter substrates of proteasome, over this time interval (Fig. 4A). To rule out alterations of the ubiquitylation pathway, cells were pre-treated with 500 nM epoxomicin (or DMSO vehicle) for 1 h before administering the high glucose challenge for 40 min. The proteasome inhibitor increased the basal content of ubiquitylated proteins in control cells and prevented their drop in high glucose treated cells (Fig. 4B).

To address whether bulk proteasome activity was induced by glucose uptake, crude cell extracts of rMC1 challenged with high glucose and low glucose or mannitol for 10, 20 and 40 min were assayed for the kinetics of Suc-LLVY-amc hydrolysis, a synthetic substrate of chymotrypsin-like activity (Fig. 4C). The cleavage rate of Suc-LLVY-amc was faster by extracts of cells challenged with high glucose at each time-point analysed (Fig. 4C). To further validate this finding, proteasome particles were separated by native-gel electrophoresis and probed with 75 µM Suc-LLVY-amc (Fig. 4D). Compared to control cells, high glucose-stimulated rMC1 displayed a robust hyper-activation of the capped assemblies (i.e., 30S–26S) which reached a peak around 20 min after stimulus delivery (Fig. 4D).

Immunostaining with an anti-Rpt5 (i.e., a 19S subunit) antibody clarified that the capped particles content was actually increased in high glucose stimulated cells with respect to control cells, in association with a drop of free 19S (Fig. 4D, *bottom panel*). To rule out uneven gel loading, crude cell extracts were further run by canonical Wb and probed with antibodies raised against α4 (i.e., 20S), Rpt5 and Rpt6 (i.e., 19S) (Fig. 4D, *bottom panel*).

Thereafter, proteasome proteolytic activity in living cells was further probed with TED, a quenched fluorogenic peptide of proteasome, according to a method validated elsewhere [32].

To meet all the chronological criteria (TED properties and timing of proteasome activation by glucose), rMC1 were first pre-treated with 500 nM epoxomicin for 1 h. Thereafter, high glucose and controls were administered and, after additional 5 min of incubation, 17 µM TED peptide was added to the culture medium and fluorescence release recorded for a total of 20 min after high glucose delivery. Following TED administration, fluorescence intensity quickly increased under all experimental conditions tested. However, after 15 min from TED addition (that is 20 min from glucose administration), whilst TED fluorescence reached a plateau in low-glucose or mannitol-treated cells, it displayed an additional steep increase in high glucose-stimulated cells (Fig. 4E).

### High glucose induces the phosphorylation of the Rpt6 subunit of the 19S

The rapidity through which proteasome activation took place was suggestive of a post-synthetic modification, such as phosphorylation, on some proteasome subunits.

**Fig. 5:**
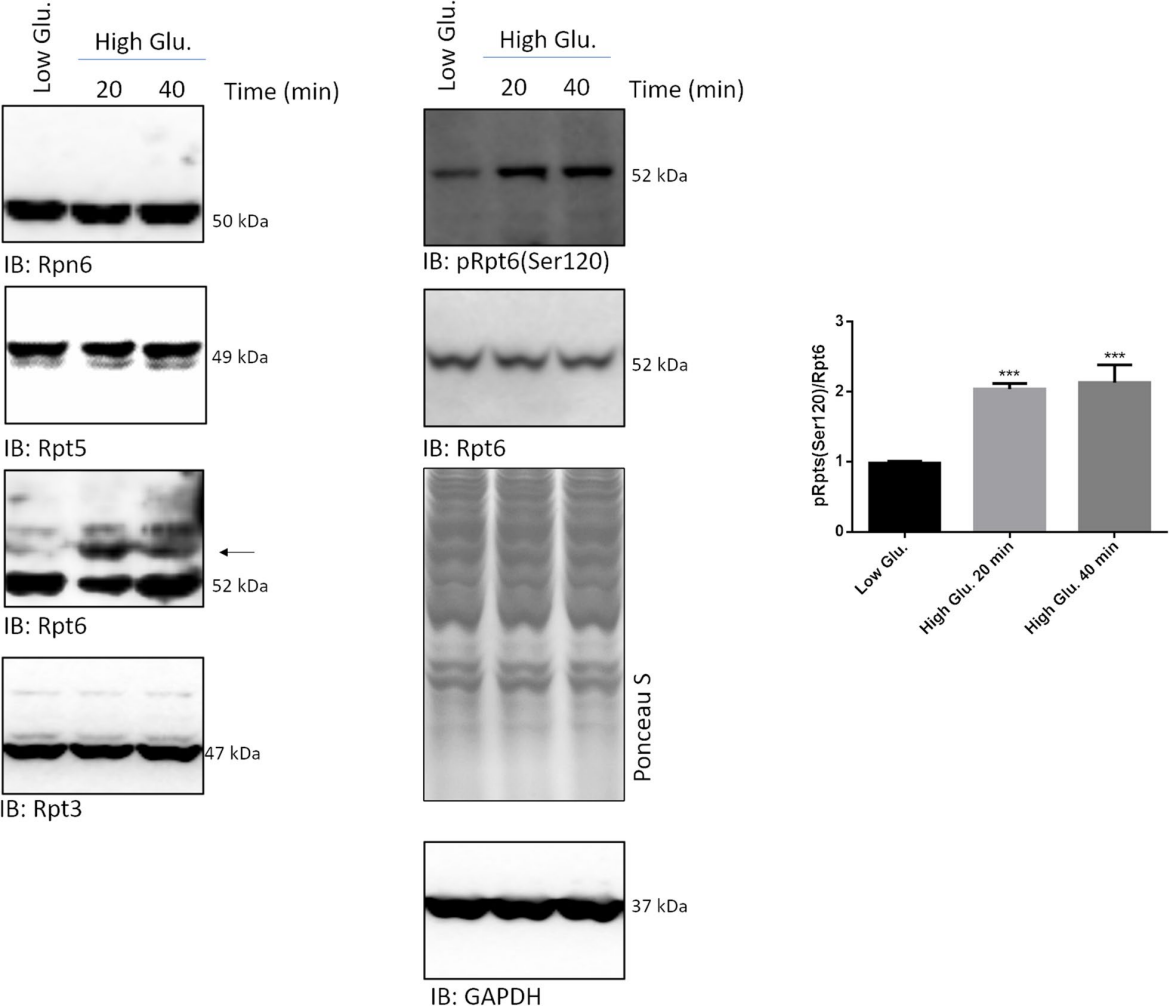
Proteasome activation is triggered by phosphorylation of Rpt6 at Serine 120. Whole cell extracts were obtained from rMC1 challenged with 25 mmol/L glucose and low glucose or mannitol for 20 and 40 min and analysed by phos-Tag assay and Wb. Phos-Tag filters were probed with antibodies anti-Rpn6, -Rpt5, -Rpt3 and -Rpt6. Rpn6 and Rpt5 blots are purposely shown overexposed to highlight the lack of species with delayed electrophoretic mobility; notably, Rpt5 migrated as a doublet band also in denaturing and reducing conditions (see Fig. 4). Wb filters were further probed with a phospho-specific antibody raised against Rpt6 phosphorylated at Serine 120 [pRpt6(Ser120] and with an anti-Rpt6 antibody. The pRpt6(Ser120)/Rpt6 ratio between immunoreactive bands was then calculated and reported in histograms. Total proteins stained by Ponceau S were used as loading control. Data are presented as mean ± SD (n = 3); a representative blot is shown. One-way ANOVA followed by Tukey’s post-hoc test: ***p < 0.001

To test this possibility, a phos-Tag assay was set up to screen the electrophoretic mobility of a panel of 19S subunits for which phospho-sites have been described, such as Rpn6, Rpt3, Rpt5 and Rpt6 [33–37], in rMC1 stimulated with 25 mmol/L glucose and controls for 20 and 40 min. Rpn6 and Rpt5 immunostaining did not reveal any delayed mobility of species others than the unphosphorylated one (Fig. 5). Rpt6 immunostaining highlighted a delayed mobility of two bands (black arrow), which, indeed, were nicely detectable under all the experimental conditions. However, these phosphorylated species stained much stronger in high glucose-treated than in controls-treated cells, and this was paralleled by a drop of the intensity of the unphosphorylated species. Conversely, Rpt3 showed delayed-mobility species but no altered ratio between phospho- and unphosphorylated bands under any of the experimental conditions tested (Fig. 5).

Whole cell lysates were then probed with a phospho-specific antibody raised against Rpt6 phosphorylated at Serine 120, a major phospho-site. Immunodetection of phospho- and unphospho-Rpt6 confirmed the presence of a significant basal level of phosphorylation of this subunit under all experimental conditions, but, compared to untreated rMC1, further highlighted a robust increase of the pRpt6(Ser120)/Rpt6) ratio in cell lysates harvested after 20 and 40 min of high glucose stimulation (Fig. 5).

### CamKII but not PKA phosphorylates Rpt6 and stimulates bulk proteasome activity in rMC1

**Fig. 6:**
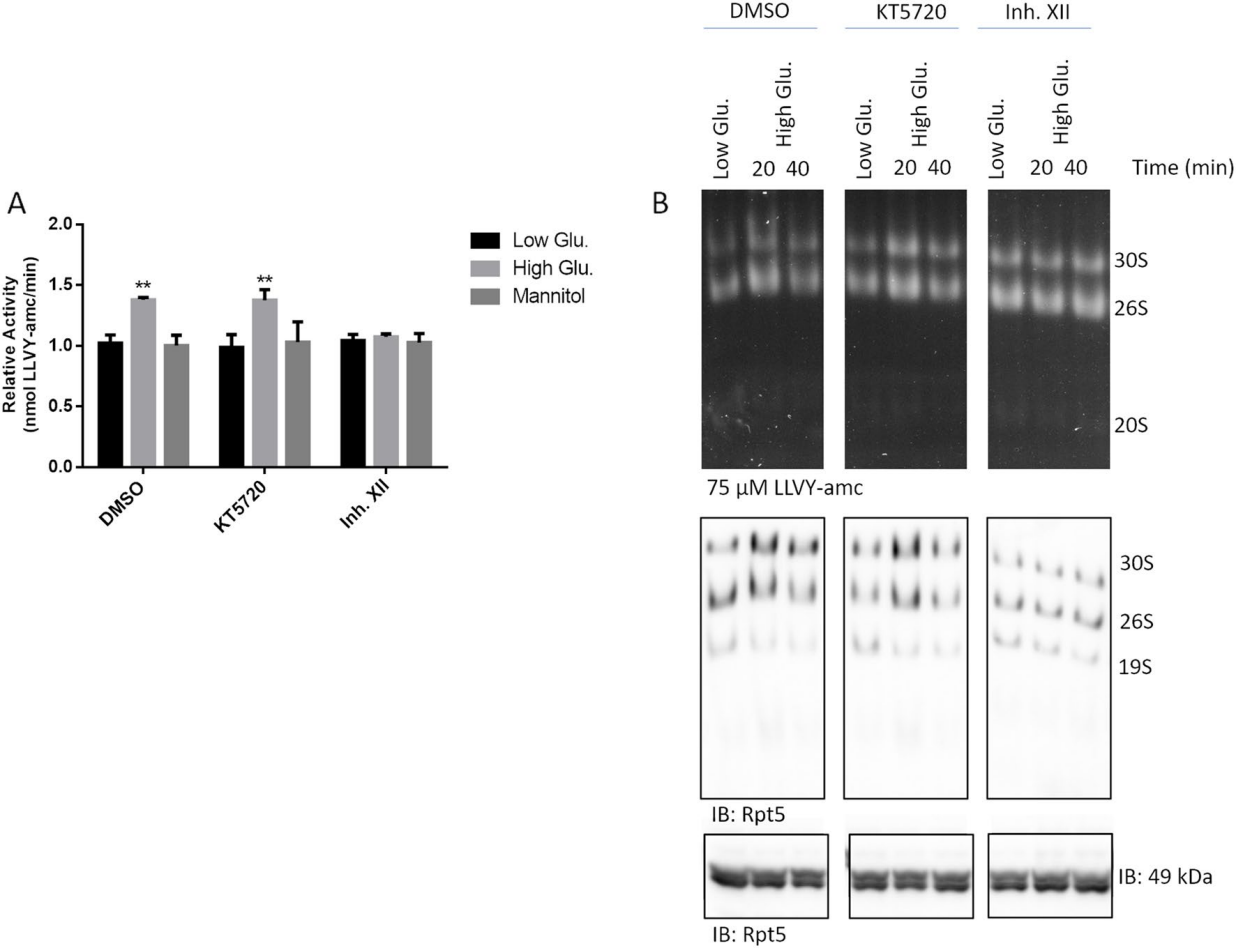

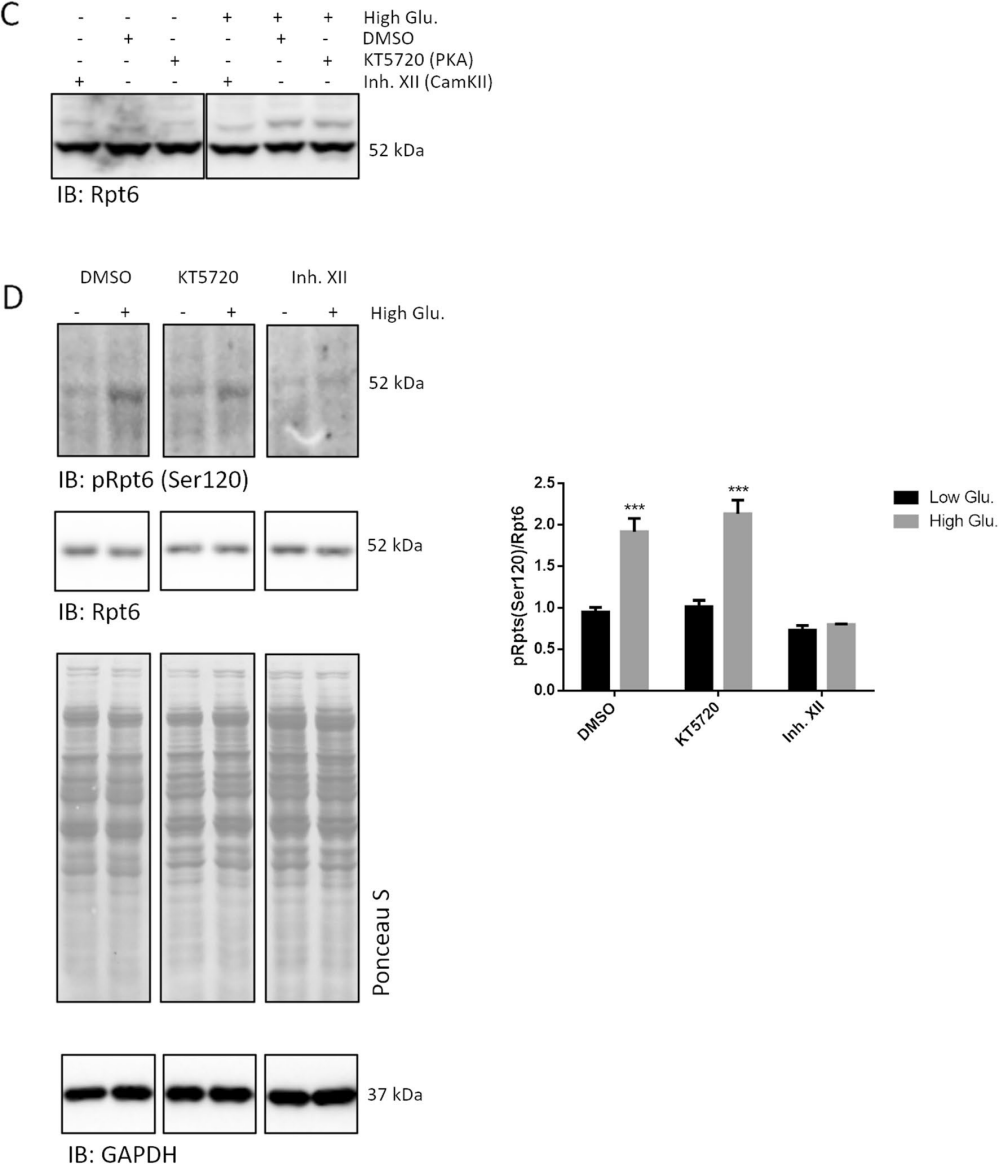
CamKII, but not PKA, phosphorylates Rpt6. **A** rMC1 cells were pre-treated with 10 µM DMSO, Inh. XII or KT2750 for 2 h. Thereafter, compounds were washed out and 25 mmol/L glucose or low glucose/mannitol was added to culture media for 20 min and crude cell extracts were prepared. Cells treated with low glucose and mannitol were used as controls. Bulk chymotrypsin like activity was determined as described in Fig. 4C and results expressed as relative activity respect to the slope of Suc-LLVY-amc cleavage by proteasome in crude cell extracts of low glucose stimulated cells. One-way ANOVA followed by Tukey’s post-hoc test (n = 3): **p < 0.01. **B** The same crude cell extracts were also run by native-gel electrophoresis (as in Fig. 4D) and the filters probed with the anti-Rpt5 antibody. An aliquot of the sample was run by denaturing and reducing Wb to rule out uneven gel loading (bottom panel). **C**, **D** Whole cell extracts were obtained from rMC1 challenged with 25 mmol/L for 20 and analysed by phos-Tag assay (**C**) and Wb (**D**). Phos-Tag filters were probed with the anti-Rpt6 antibody. Wb filters were probed with the phospho-specific anti-Rpt6(Ser120) antibody and with the anti-Rpt6 antibody. The pRpt6(Ser120)/Rpt6 ratio was then calculated from immunoreactive bands of high glucose vs low glucose lanes and shown in histograms. Total proteins stained by Ponceau S were used as loading control. Data are presented as mean ± SD (n = 3), a representative blot of three independent replicates is shown. One-way ANOVA followed by Tukey’s post-hoc test: ***p < 0.001

To identify the kinase that actually phosphorylated Rpt6, we first focused on the calcium-dependent calmodulin Kinase II (CamKII) and protein kinase A (PKA) by using two specific inhibitors called Inhibitor XII (Inh. XII) and KT5720, respectively. First, we ruled out the presence of major effects of the inhibitors on proteasome activity under basal growth condition (Additional file 1: Fig. S2). Then, after a 2 h pre-treatment with the two inhibitors (each delivered at 10 µM) (or DMSO vehicle), rMC1 cells were stimulated with 25 mmol/L glucose or controls for 20 min and crude cell extracts probed for the kinetics of Suc-LLVY-amc hydrolysis (Fig. 6A). Cells treated with DMSO or KT5720 displayed an increase of Suc-LLVY-amc hydrolysis by high glucose comparable to that reported previously (see Fig. 4B) (Fig. 6A). Conversely, Inh. XII robustly opposed proteasome activation at 20 min of the high glucose challenge (Fig. 6A).

This finding was confirmed also by native-gel electrophoresis (Fig. 6B). Again, whilst DMSO and KT5720 did not alter the proteolytic activation of the capped assemblies by 25 mmol/L glucose (see Fig. 4D), Inh. XII prevented particle activation by sugar intake, both at 20 and 40 min after challenge induction (Fig. 6B). Interestingly, unlike DMSO and KT5720, Inh. XII further reduced the engagement of the free 19S in assembling the capped particles in the presence of 25 mmol/L glucose (Fig. 6B).

Thereafter, the phos-Tag approach confirmed that Inh. XII blocked the phosphorylation of Rpt6, whilst DMSO and KT5720 did not, as the delayed-mobility band staining positive for Rpt6 was increased only in the combined presence of the two latter compounds and of high glucose (Fig. 6C).

Moreover, in cells challenged with high glucose, the immunodetection of pRpt6(Ser120), as well as the pRpt6(Ser120)/Rpt6 ratio were markedly more robust in DMSO and KT5720 treated-cells than in Inh. XII-treated cells which further displayed a drop of basal phosphorylated Rpt6 (Fig. 6D).

To further verify that Rpt6 is actually phosphorylated at Serine 120 by CaMKII in rMC1, we assayed the immunodetection of this phospho-site after silencing the *CaMKIIα* gene, an isoform already studied in this cell model for its involvement in the signalling cascade of high glucose stimulation, by delivery of 27-mer siRNAs [9]. To this aim, rMC1 grown in standard low glucose medium were challenged with two individual siRNAs (referred to as siRNA#1-#2) or a pool of non-targeting siRNA (referred to as Pool) for 72 h. As internal control, rMC1 were also left untreated (referred to as Ctrl). Whole cell lysates were then harvested and analysed by Wb to verify effective silencing of CaMKIIα and pRpt6(Ser120) content (Additional file 1: Fig. S3).

With respect to Ctrl cells and cells challenged with the non-targeting pool, both siRNA#1 and siRNA#2 induced a significant downregulation of kinase content, although to a slightly different extent (siRNA#2 > siRNA#1) (Additional file 1: Fig. S3).

Interestingly, pRpt6(Ser120) immunodetection paralleled that of CaMKIIα under all experimental conditions tested. Thus, with respect of Ctrl cells, a significant drop of the basal phosphorylation of Rpt6 was documented in the presence of siRNA#1 and siRNA#2, but not in the presence of the non-targeting Pool (Additional file 1: Fig. S3). Conversely, basal level of unphosphorylated Rpt6 were unaltered by treatment. Hence, with respect to Ctrl and Pool-treated cells, the pRpt6(Ser120)/Rpt6 ratio did significantly drop in siRNA#1 and siRNA#2 treated cells.

### Inhibition of CamKII and proteasome blocks NF-kB activation by high glucose in rMC1

We then tried to address whether proteasome and NF-kB activation were actually part of the same pathway.

First, we investigated the effects of Inh. XII, KT5720 and epoxomicin on the canonical NF-kB signalling and on CamKII content over 2 h of stimulation under basal growth conditions (Additional file 1: Fig. S4). Whole lysates were analysed by Wb revealing no alteration of IkBα and IKKβ and CamKII content (Additional file 1: Fig. S4). Immunostaining of pIkBα(Ser32), was unaltered by Inh. XII and KT5720 but displayed a robust increase at 2 h of epoxomicin stimulation.

**Fig. 7:**
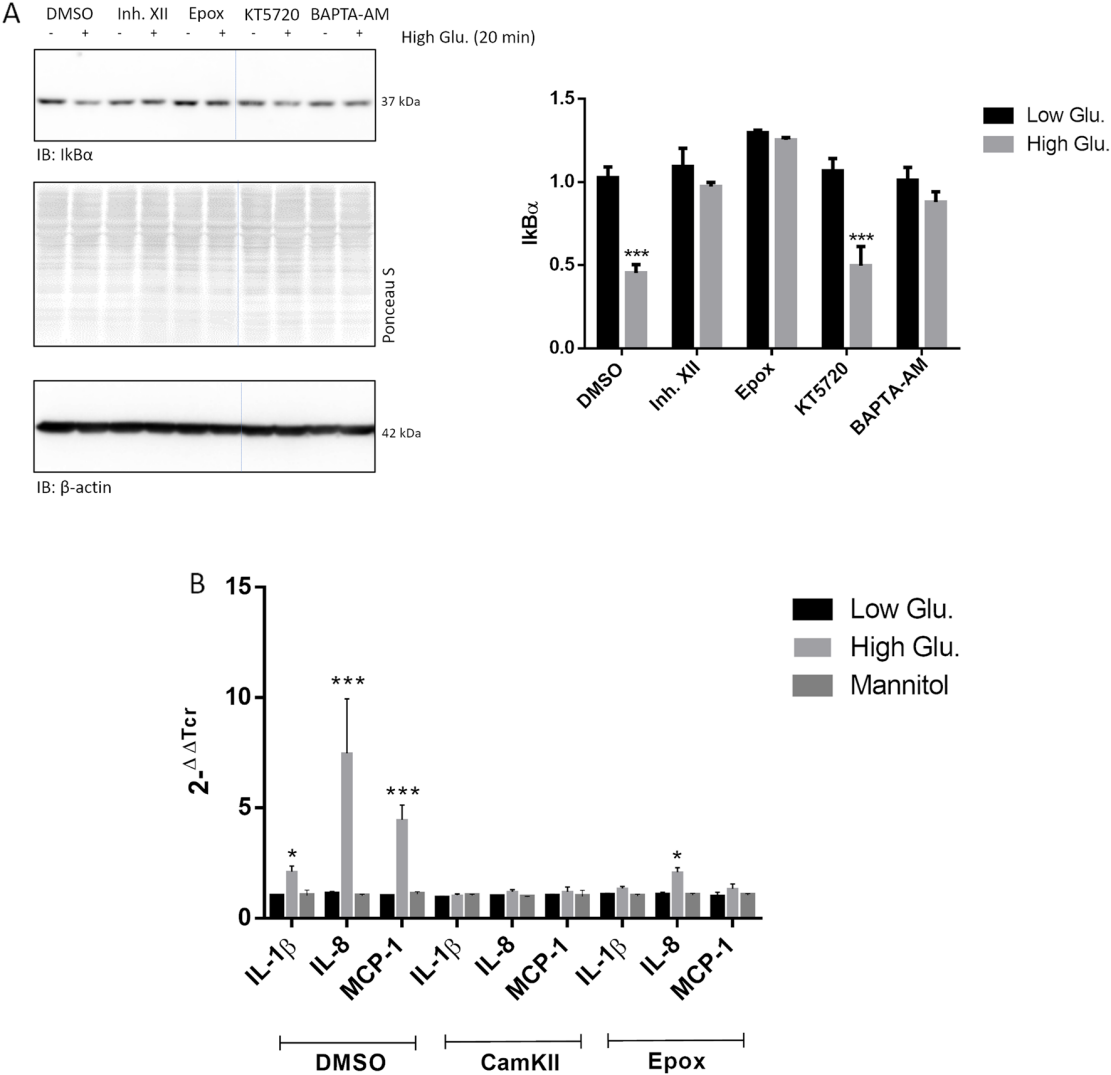
Treatment with CamKII and proteasome inhibitors prevents NF-kB from being activated by high glucose. **A** rMC1 cells were pre-treated with 10 µM DMSO, Inh. XII, KT570 or BAPTA-AM for 2 h and with 500 nM epoxomicin for 1 h. Thereafter, compounds were washed out and 25 mmol/L glucose and low glucose/mannitol were added to culture media. After 20 min, whole cell lysates were prepared and analysed by Wb. Filters were probed with the anti-IkBα antibody. Total proteins stained by Ponceau S were used as loading control. Band intensity is reported in histograms. Data are presented as mean ± SD (n = 3); a representative blot is shown. Statistics was calculated by comparing each pair low–high glucose for any given compound assayed Multiple τ Student’s tests ***p < 0.001; The bands shown belong to the same gel, but this figure has been manipulated to clear out two lanes which were unnecessary for discussion (see uncropped files). **B** rMC1 cells were pre-treated with 10 µM DMSO and Inh. XII for 2 h and 500 nM epoxomicin for 1 h. Thereafter, compounds were washed out and 25 mmol/L glucose and low glucose or mannitol (controls) were added. After 40 min of stimulation nucleic acids were isolated and the expression of pro-inflammatory cytokines assayed by RT-PCR. β-actin was used as internal control. Histogram represents the relative quantity of transcripts calculated for any given cytokine for each experimental group (i.e., low glucose, high glucose and mannitol). Statistics was calculated for each cytokine separately. Data are presented as mean ± SD (n = 3). One-way ANOVA followed by Tukey’s post-hoc test (n = 3): *p < 0.05; ***p < 0.001

Then, the turnover of IkBα after 20 min of high glucose challenge was investigated after having pre-treated rMC1 with each inhibitor. BAPTA-AM (10 µM), a validated cell-permeable Ca^2+^ chelator was also tested in this study. With respect to control cells, induction of IkBα decrease by high glucose was documented only in the presence of DMSO and of KT5720. Addition of either Inh. XII, epoxomicin or BAPTA-AM blocked the degradation of IkBα (Fig. 7A). To figure out whether inhibition of CamKII by Inh. XII and of proteasome by epoxomicin was effective also in inhibiting the transcriptional upregulation of pro-inflammatory cytokines, IL-8, IL-1β and MCP-1 transcripts were assayed by RT-PCR in rMC1 cells exposed or not to 25 mmol/L glucose for 40 min (Fig. 7B). Pre-treatment with Inh. XII or epoxomicin almost abolished the upregulation of the cytokines by high glucose (Fig. 7B) while DMSO had no effect (see comparison with Fig. 1B).

**Fig. 8:**
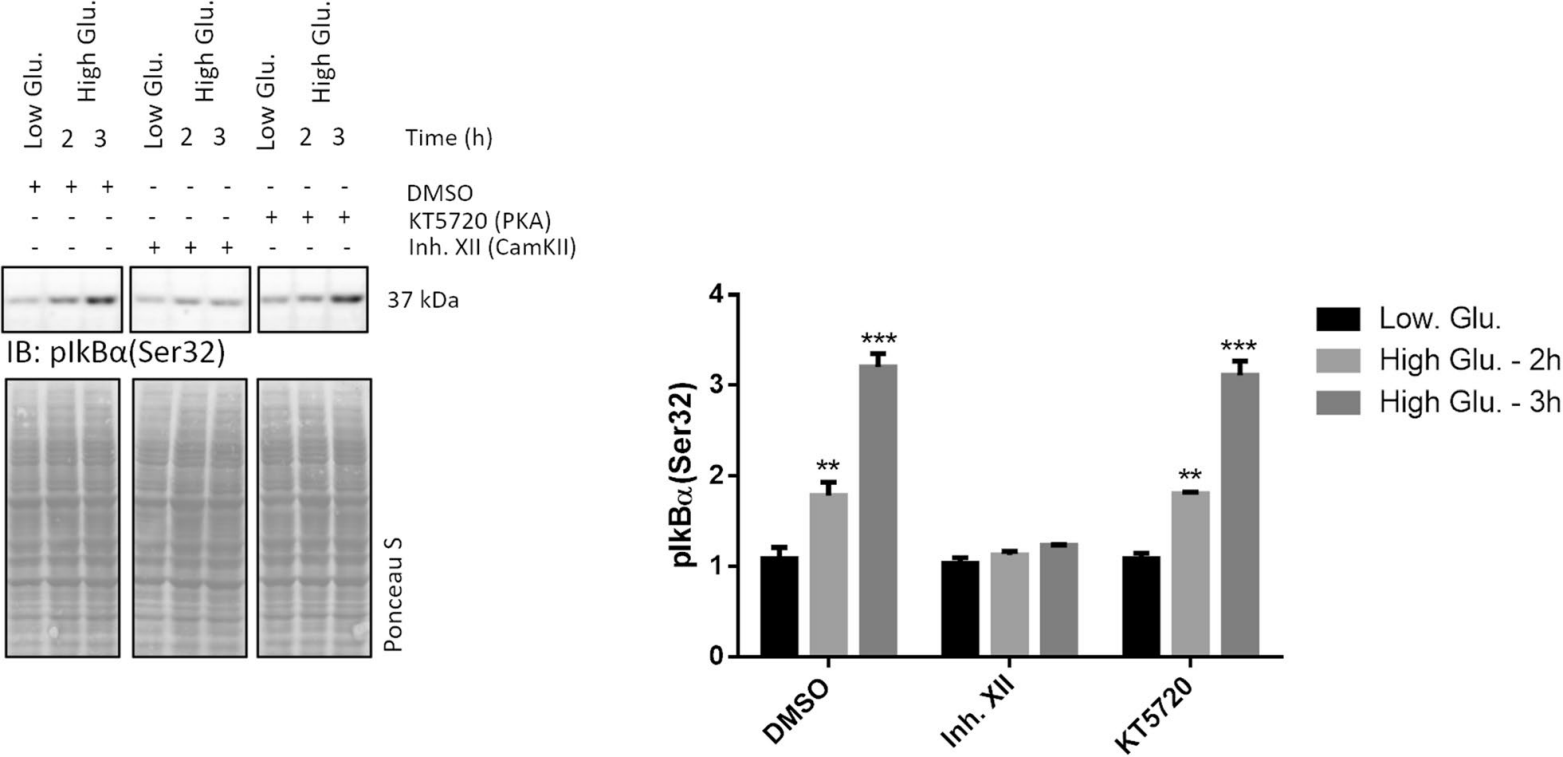
Inh. XII, but not KT5720, prevent canonical signalling of NF-kB from being activated after 2 h of high glucose challenge. rMC1 cells were pre-treated with 10 µM KT5720, Inh. XII or DMSO for 2 h. Thereafter, 25 mmol/L glucose or the same volume of 5 mmol/L glucose was administered. Whole cell lysates were prepared at 2 and 3 h after glucose delivery and analysed by Wb. Filters were probed with the anti-IkBα(Ser32) antibody. Remarkably, epoxomicin was not enrolled due to increase of pIkBα(Ser32) at these time-points as indicated in Additional file 1: Fig. S4. Total proteins stained by Ponceau S were used as loading control. A representative experiment of three independent replicates is shown. Histogram represents the relative levels of pIkBα(Ser32) protein in the different experimental conditions. One-way ANOVA followed by Tukey’s post-hoc student test: **p < 0.01, *** < p.0.001

To test whether CamKII, but not PKA inhibition was effective also in halting pIkBα phosphorylation at Serine 32 during the second cycle of NF-kB activation (see Fig. 2), rMC1 cells, pre-treated with Inh. XII and KT5720 were challenged with high glucose for 2 and 3 h (Fig. 8). Compared to control cells, pIkB(Ser32) immunostaining in the presence of high glucose was increased in cells treated with DMSO or KT5720, whereas Inh. XII drastically opposed the accumulation of this phosphorylated protein (Fig. 8).

### Influence of Rpt6 mutagenesis on early NF-kB activation in rMC1 exposed to high glucose

To clarify the role of Rpt6 phosphorylation in driving the early NF-kB activation, rMC1 were stably transfected with a plasmid encoding a phospho-dead mutant Rpt6 in which Serine 120 was replaced by alanine (hereafter referred to as Rpt6_S120A) and with the empty vector (hereafter referred to as Vector) as internal control. Two independent clones for Vector and Rpt6_S120A were generated.

Following transfection, the Rpt6 immunostaining was greater in Rpt6_S120A than Vector cells, whilst that of additional Rpt(s) subunits was not, and pRpt(Ser120) was significantly reduced in mutated clones (Additional file 1: Fig. S5A).

Surprisingly, Rpt6_S120A clones displayed a twofold increase of chymotrypsin-like activity on Suc-LLVY-amc with respect to Vector clones (Additional file 1: Fig. S5B). Furthermore, the capped assemblies and, most notably, the free 20S of Rpt6_S120A clones were significantly more active than those isolated from Vector cells, as from native-gel studies (Additional file 1: Fig. S5C).

Probing of filters with an anti-Rpt3 antibody made it clear that the immunostaining of capped particles was markedly increased, especially that of the 30S, whereas the free 19S content was significantly reduced in Rpt6_S120A vs Vector clones. Conversely, the 20S immunostaining by an anti-α4 antibody was unaltered between clones, implying that the free 20S of Rpt6_S120A clones was hyperactive (Additional file 1: Fig. S5D).

Since the mass/charge electrophoretic pattern of the free 20S argued against the binding of an alternative regulatory particle [38, 39], to interpret the 20S hyperactivation, whole cell lysates of all clones were run in parallel by phos-Tag and Wb. Filters were probed with an anti-α4 and an anti-pan-α20S antibody, that targets 6 out of 7 α-subunits except for α4, producing a typical smear. The phos-Tag approach highlighted (Additional file 1: Fig. S5E) that a delayed-mobility species of α4 was missing out in Rpt6_S120A clones in the absence of variation of total α4 (Additional file 1: Fig. S5E). By probing the same filters with the pan-α20S antibody it emerged that the pattern of the six subunits was unaltered, although in the former case separation of individual subunits was unsuccessful probably due to the presence of constitutive phosphorylation of some subunits which altered the migration pattern superimposing with that of at least some of them (Additional file 1: Fig. S5E).

To address the consequences of proteasome activation on the dynamics of NF-kB, a panel of proteins involved in the pathway were assayed by Wb. Surprisingly, IkBα displayed a robust drop in Rpt6_S120A clones (Additional file 1: Fig. S6A). Conversely, pIkBα(Ser32) was significantly increased and p65 unaltered (Additional file 1: Fig. S6A). Yet, the poly-ubiquitinylated proteins were slightly and not significantly increased in the Rpt6_S120A clones together with p21, whilst p53 was significantly increased in these cells (Additional file 1: Fig. S6B).

Based on the IkBα results, the transcription of pro-inflammatory cytokines was then assayed by RT-PCR. Surprisingly, the IL-8 mRNA was found to be nearly fivefold higher in Rpt6_S120A vs Vector clones, whereas MCP-1 did show a nearly 3.5-fold increase (Additional file 1: Fig. S6C). IL-1β (*data not shown*), instead, was characterized by a significant inter-experimental variability which did not allow to draw unambiguous conclusions.

## Discussion

Herein, we describe an early and atypical pathway of p65-p50 transcriptional activation by high glucose in rMC1 whose distinctive feature is the lack of any obvious IkBα phosphorylation, seemingly replaced by the CamKIIα-dependent phosphorylation of the 19S Rpt6 subunit. Furthermore, we have confirmed the involvement of NF-kB canonical pathway in rMC1 pro-inflammatory polarization, but we provide evidence that, at least under the experimental conditions tested, this pathway is triggered much earlier than originally thought.

Muller glia cells undoubtedly play a significant role in the pathological retinal remodelling of diabetic subjects, as this cell type has been continuously reported to acquire morpho-functional markers of activation in the presence of high glucose concentrations and, notably, to express and secrete a plethora of pro-inflammatory cytokines and neurotransmitters [5, 7, 40, 41]. Remarkably, by virtue of their anatomical and functional proximity, MG, pericytes and endothelial cells, are supposed to constitute a neurovascular unit inside which the metabolic alterations are readily transduced across the cell lineages [42].

NF-kB is a highly conserved pro-inflammatory pathway which serves crucial biological activities for neuron homeostasis [11, 14, 15]. In the canonical pathway, p65-p50 transcriptional activation is dependent on a thermodynamic equilibrium between the availability of free IkBα, bound IkBα, the rate of its phosphorylation by pro-inflammatory signalling cascades and kinetics of proteasome clearance [11]. Biochemical and mathematical studies have provided elegant evidence of how the artificial increase of free IkBα half-life, through mutagenesis of the PEST sequence, turns out into a reduced rate of p65-p50 activation [11].

In this study, NF-kB activation appears to be strictly dependent on CamKII and proteasome, since inhibition of either one of these two enzymatic activities led to a blockade of the pathway.

Whilst the occurrence of CamKIIα activation by Ca^2+^ quickly after high glucose delivery is supported by previous studies in rMC1 which highlighted the involvement of P2X7 purinergic receptors in the dynamics of Ca^2+^ flux, the contribution of proteasome appears to be currently drawn only through thermodynamic assumptions [43].

Bulk proteasome activity is supposed to be very low in comparison with the effective concentration of assembled particles, as they mostly lie in a substrate-engagement competent but proteolytic-incompetent state in living neurons [44]. The rate-limiting step of substrates degradation through the UPS has been long considered to be the Ub conjugation by E3-ligases, but Ub-tagged substrates are thought to bind constantly to catalytic-incompetent proteasome assemblies without being digested [30]. In this framework, proteasome phosphorylation is a post-synthetic mechanism which quickly increases the pool of catalytic-competent particles and the velocity of poly-ubiquitylated short-lived substrates processing, orienting the kinetics of metabolic pathways regulated through targeted proteolysis [19, 31, 33, 35, 37, 45].

In this regard, NF-kB and the kinetics of IkBα binding and dissociation with p65-p50 represent an ideal thermodynamic equilibrium by which the enzyme (e.g., proteasome) hyper-activation subtracts the bioavailability of a reactant, in this case (we hypothesize) the constitutively phosphorylated IkBα, which is readily ubiquitylated and digested, shifting the whole equilibrium toward IkBα consumption and p65-p50 release. We must suppose that proteasome hyperactivation pushes forward the IkBα-pIkBα equilibrium, serving as a kinetic activator of a pathway regulated by the dissociation rate constant of IkBα, which indeed occurs in few minutes [46]. Although it may be determined by additional factors, the 10 min delay between pIkBα(Ser32) and IkBα decrease after high glucose delivery may represent a further clue for this thermodynamic-driven equilibrium. [47, 48]. However, it is also possible that the 26S proteasome degrades free IkBα in the absence of ubiquitylation, but the equilibrium would proceed as well toward the direction proposed. Nevertheless, a possible involvement of phosphatases in decreasing the pIkBα(Ser32) content is unlikely, as this occurrence would reasonably promote IkBα stabilization and retention of p65-p50 into the cytosolic compartment.

Although speculative, the synthesis and secretion of pro-inflammatory cytokines during the first round of transcriptional activation would act as an autocrine and paracrine stimulus for cyclic NF-kB activation which follows a canonical pro-inflammatory pathway. Nevertheless, the inhibition of the second round of NF-kB activation obtained through inhibition of CamKII, may indirectly support this possibility, although, at this stage, it cannot be ruled out that additional pathways, regulated by CamKII, might be responsible for this phenomenon. It is further worth pointing out that the pattern of cytokines transcribed is suggestive of a tailored regulation of p65-p50 activity, probably through post-synthetic modifications, as IL-6 and TNF-α, which are commonly induced together with IL-1β, in this case were not [34, 49]. However, it must be pointed out that only a full characterization of the NF-kB pathways herein described at 45 min and 3 h by high-throughput strategies (e.g., RNAseq) can definitively address how the pattern of pro-inflammatory polarization of rMC1 is regulated over this brief time interval.

The introduction of the Rpt6_S120A phospho-dead mutation is likely followed by a metabolic adaptation of the cells which involves both the capped assemblies, and most notably, the free 20S, which was a very unexpected finding also because the same mutation was reported to decrease basal proteasome activity in other eukaryotic systems [34, 49]. Nonetheless, it is worth pointing out that Rpt6 looks to be phosphorylated at a significant basal level in rMC1, suggesting that this post-synthetic modification is of great relevance for the metabolism, at least for this cell type, and that its absence induces paradoxical effects through compensatory mechanisms. One of them may involve the phosphorylation of α4 subunit, which has been previously reported to inhibit 20S activation [50]. In this respect, while this manuscript was being written, transgenic mice harbouring the Rpt6_S120A mutation displayed minimal defects on synaptic plasticity and learning, in spite of previous evidences ex vivo that these brain processes were regulated through this PTM, suggesting that this mutation might activate still unknown compensatory mechanisms [51].

Thus, we very preliminarily hypothesize that phosphorylation of Rpt6 could be the specific PPTM coupling high glucose stimulation and proteasome activation of rMC1. However, this could represent one out of different proteasome PTMs serving roles for NF-kB regulation and it would be of broader biological relevance to figure out whether modulation of proteasome activity, regardless of the mechanism (e.g., PTMs, drugs, etc.) through which it is reached out, foster alternative signalling networks of pathways regulated by targeted proteolysis, such as NF-kB; this possibility was somewhat theorized by the “load vs capacity” model [52]. In this framework, it is worth recalling that proteasome-dependent regulation of inflammation in diabetes onset and progression has been already envisaged. The clearance of amyloidogenic hIAPP peptide in Langherans islets through the UPS has been tightly linked to inflammasome activation and loss of β-cells [53, 54].

In conclusion, if this molecular scenario here depicted will be confirmed by additional experimental of greater complexity in ex vivo and in vivo models, it may serve as a clue for hypothesizing therapeutic approaches which exploits proteasome hyperactivation increasing the rate of degradation of selected targets through non-conventional drugs such as Proteolysis Targeted Chimeras (PROTACs).

## Materials and methods

### Cells and cell culture conditions

The immortalized rat retinal Müller cells (rMC-1) were obtained from Kerafast (Kerafast, Boston, MA, USA) [55] and were maintained in 5 mmol/L glucose medium (Low Glucose DMEM), supplemented with 10% FBS plus antibiotics (Lonza, Basel, Switzerland). The challenge with 25 mmol/L glucose (referred to as high glucose throughout the text) was performed by delivering either High Glucose DMEM, or Low Glucose DMEM supplemented with D+ Glucose powder (20 mmol/L). The two treatments provided fully overlapping results. Hyperosmolar stress was induced by delivering Low Glucose DMEM supplemented with 20 mmol/L mannitol. Remarkably, data from low glucose and mannitol treated cells were fully overlapping, confirming that osmolarity does not affect rMC1 activation, as commonly described by several authors [7–9, 43]. Thus, for the sake of readership, in the case of Wb analysis, data of low glucose and mannitol-treated cells are not discussed separately, unless otherwise indicated, and are generally referred to as untreated/control cells. In all cases, stimuli were delivered on cells grown at 70% confluency.

### Proteasome assay, native gel electrophoresis and TED fluorescence in vivo

Cells were lysed under non-denaturing conditions by freeze-thawing cycles in 250 mM sucrose, 20% glycerol, 25 mM Tris–HCl, 5 mM MgCl_2,_ 1 mM EDTA, 1 mM DTT, 2 mM ATP, pH 7.4, as described elsewhere [56, 57]. Thereafter, lysates were cleared by centrifugation at 13.000 rpm, 20 min, 4 °C and protein concentration determined by Bradford assay.

For proteasome assays, 20 µg of proteins were diluted in 20% glycerol, 25 mM Tris–HCl, 5 mM MgCl_2,_ 1 mM EDTA, 1 mM DTT, 2 mM ATP, pH 7.4 in the presence or absence of 500 nM epoxomicin in a Corning 96-well Black Microplate. Reaction mixtures were pre-incubated 30 min at 37 °C. Thereafter, 50 µM 7-amino-4-methylcoumarin (AMC) labeled Suc–Leu–Leu–Val–Tyr–AMC peptide (referred to as Suc-LLVY-amc) (Boston Biochem, Boston, MA, USA) was delivered to each well and the release of fluorescence monitored over 40 min–1 h (in any case, until linearity was observed) in a Cary Eclipse Varian spectrofluorimeter. Values obtained, representative of the moles of substrate processed per time (indicated as nmol substrate/min) were calculated and plotted. The rate of peptide hydrolysis (negligible) in the presence of epoxomicin was subtracted from that in the absence of the proteasome inhibitor. Each experimental condition was run in triplicate into the same plate. The slopes of each curve were then figured out and compared at each time-point.

For native-gel electrophoresis, 75 µg of proteins from each experimental condition were separated under native conditions into a 3.5% polyacrylamide gel. Gels were then soaked in in reaction buffer (50 mM Tris, 5 mM MgCl_2_, 1 mM ATP, pH 7.5) supplemented with 75 µM Suc-LLVY-amc. Complexes were then transferred to a HyBond-ECL nitrocellulose filters (Amersham Biosciences, Amersham, UK) after a mild denaturation in 1% SDS 25 mM Tris–HCl and probed with an anti-Rpt5, -Rpt3 (Protein-tech Group, Manchester, UK), or -α4 (EnzoLife Science, London, UK) antibodies, diluted 1:3000 in 0.02% Tween-PBS plus fat-free milk and, thereafter, incubated with a HorSeradish Peroxidase-conjugated anti-rabbit or anti-mouse IgG antibody (Biorad, Hercules, CA, USA), diluted 1:50000 in 0.2% Tween-PBS fat-free milk.

The release of TED (a generous gift of prof. Mario Salmona, IRCCS Mario Negri, Milan, Italy) fluorescence by living rMC1 was determined as previously described [32]. Briefly, cells were pretreated with 500 nM epoxomicin or equivalent volume of DMSO for 1 h. The compounds were washed out and high glucose or control media were delivered. After 5 min of incubation 17 µM TED was added to the cell culture and fluorescence release recorded in a Tecan Spark spectro-fluorimeter over 20 min from delivery.

### Western blotting

For denaturing and reducing western blotting (Wb), cell pellets were processed through different approaches depending on the target.

For unphosphorylated proteins, cell pellets were lysed in RIPA buffer supplemented with cocktails of proteases and phosphatases inhibitors and cleared by centrifugation at 13000 rpm for 30 min, at 4 °C. Protein concentration was determined by Bradford assay. Depending on the target, 2 up to 40 µg of proteins per lane were loaded.

For phosphorylated proteins analysis, including experiments of CaMKIIα silencing (see below), cells were directly lysed in 2 × Laemmli buffer supplemented with 1 mM DTT, vortexed 1 min at maximum speed and heat-denatured 10 min at 95 °C. This lysis method was preferred also for analysis through phos-Tag polyacrylamide gels performed following manufacturer’s instructions (Wako Films, Chemicals, Richmond, VA, USA). Notably, phos-Tag acrylamide refers to an electrophoretic procedure based on a synthetic acrylamide which delays the electrophoretic migration of phosphorylated proteins. Protein separation is followed by canonical Wb procedure which allows to stain the phosphorylated and unphosphorylated species of any given protein.

Different acrylamide concentrations were tested and the best results were obtained by using 15% polyacrylamide-gels supplemented with 50 µM of phos-Tag reagent.

Canonical western blotting was set up depending on the target: a range of 10%-15% polyacrylamide gels were used. A 15% composition furnished better quality images in the case of pRpt6(Ser120). In this case, Ponceau S staining was not performed, as this treatment (probably, Ponceau de-staining with strong bases) was shown to impair protein detection. Conversely, pIkBα(Ser32) immunostaining was unaffected by Ponceau staining. Whilst blocking procedure was generally performed as described in the previous section, that of filters probed with the anti-Rpt6(Ser120) antibody (Mybiosource, San Diego, CA; USA) were processed as it follows: non-specific binding sites were saturated with a 2.5% albumin (Sigma-Aldrich, St- Louis, Co, USA) and 2.5% fatty-free milk dissolved in Tris–HCl Buffered Saline (TBS) pH 7.5 plus 0.05% Tween for 3 h at room temperature. The antibody was diluted 1:1000 in the same solution, and incubated overnight at 4 °C. Subsequent procedures were performed as previously indicated. Antibodies used for NF-kB signalling pathway and for CamKII immunodetection were all purchased from Cell Signalling (London, UK). The antibody named pan-α20S was purchased from EnzoLife Science (London, UK). Antibodies raised against cytokines were purchased from Abclonal (Woburn, MA, USA).

As a general note for the Western blotting analysis herein studied, since preliminary settings showed that, in most cases, the micrograms loaded fall off the dynamic linear range of canonical antibodies used as internal controls (e.g., tubulin, actin, GAPDH), even though internal controls lanes are generally reported, normalization was performed on Ponceau S staining unless otherwise indicated (see Additional file 1: Fig. S7). The choice of actin or GAPDH was dictated by the overlapping molecular weights of these internal controls with the proteins here studied (IkBα and GAPDH show similar MW).

Densitometric analysis of the bands was performed through ImageJ Quant Software. Unless otherwise indicated (see Fig. 7), original figures manipulation was limited to adjustments of light/contrast by using the specific command or the “curve” tool of GIMP2.0 software.

### Gene expression analysis

RNA was isolated with Trizol reagent (Life-tecnologies). First strand cDNAs were synthesized from 1 µg of total RNA in a 20 µl reaction with reverse transcriptase according to manufacturer instructions (BioLine, London, UK). Real-time PCR was performed on 30 ng of cDNA, using a SYBR green Master Mix (Biorad, Hercules, CA, USA). β-actin was used as internal control. All primers used in these experiments are reported in Table 1.

**Table 1:** List of primers used for RT-PCR analysis and of siRNAs for silencing experiments

Gene	Sequence
*β-actin*	5ʹ-AGCCATGTACGTAGCCATCC-3ʹ 5ʹ-CTCTCAGCTGTGGTGGTGAA-3ʹ
*IL-1β*	5ʹ-ACTCATTGTGGCTGTGGAGA-3ʹ 5ʹ-TAGCAGGTCGTCATCATCCC-3ʹ
*IL-6*	5ʹ-TGATGGATGCTTCCAAACTG-3ʹ 5ʹ-GAGCATTGGAAGTTGGGGTA-3ʹ
*IL-8*	5ʹ-GAAGATAGATTGCACCGA-3ʹ 5ʹ-CATAGCCTCTCACACATTTC-3ʹ
*MCP-1*	5ʹ-GCCAGTGAATGAGTAGCAAG-3ʹ 5ʹ-CTTCTGGGCCTGTTGTTCAC-3ʹ
*TNF-α*	5ʹACTGAACTTCGGGGTGATTG-3ʹ 5ʹGCTTGGTGGTTTGCTACGAC-5ʹ
*CamKIIα siRNA#1 (27mer duplex)*	rArCrArArGrArArGrArArUrGrArUrGrGrCrGrUrGrArArGrGrA
*CamKIIα siRNA#2 (27mer duplex)*	rGrGrCrCrUrGrGrArCrUrUrUrCrArUrCrGrArUrUrCrUrArTrT

### Silencing assays

For CamKIIα silencing assays, rMC1 were seeded at a density of 4–6 × 10^4^ per well on a 6-well plate in complete growth medium (low glugose) to obtain 50–70% confluence. The following day, CamKIIα siRNA (siRNA #1-#2), along with the non-targeting Pool (OriGene, Rockville, MA, USA), were delivered at 40 nM upon dilution in Dharmacon transfection medium (Dharmacon Horizon, Cambridge, UK).

After 72 h from siRNA delivery, cells were harvested by lysis in Laemmli buffer as introduced before and analysed by Wb.

### Rpt6 mutagenesis

PT-Rex-DEST30 control vector and PT-Rex-DEST30, in which Rat Rpt6 cDNA with a phospho-dead mutation Serine to alanine (S120A) was cloned, were purchased from Life Technologies (London, UK). Plasmids were re-suspended in 10 μl of sterile water to prepare 1 μg/μl stock solution and stored at – 20 ºC until use. One day before transfection, rMC1 cells were seeded at a density of 4–6 × 10^4^ per well on a 6-well plate in complete growth medium to obtain 50–70% confluence the following day. The day of transfection, 1 μg of DNA (PT-Rex-DEST30 control vector and PT-Rex-DEST30-Rpt6(S120A) was diluted into Turbofectin transfection reagent (OriGene, Rockville, MA, USA) and added to seeded cells according to manufacturer’s instructions. Then, cells were incubated for 48 h and after, cells were splitted (at 1:10 or higher dilution) into fresh growth medium containing selective medium. The selection was performed using 1000 μg/mL geneticin (Sigma Aldrich, St. Louis, MI, USA) for 2 weeks. Different resistant clones for each plasmid were selected and routinely grown in selective medium containing 300 μg/mL geneticin.

For induction experiments with tetracycline, transformed cells were seeded at a density of 7–10 × 10^4^ per well on a 6-well plate in selective medium containing 300 μg/mL geneticin. The following day, 0.5 μg/mL of tetracycline (Sigma Aldrich, St. Louis, MI, USA) was added and incubated for 24 h at 37 ºC.

### Data analysis and statistics

In all cases, values reported are expressed as mean ± SD. One-way ANOVA followed by Tukey post-hoc significance test and τ Student’s test were used to calculate statistically significant differences between groups. Unless otherwise indicated, comparisons were made between low-glucose/mannitol treated cells and high glucose treated cells. Data of Wb immunoreactive bands were presented as relative ratio and a nominal value of 1 was assigned to the first lane of the filter. To simplify the statistical analysis reported, * refers to p values between 0.05 and 0.01, ** to p values between 0.01 and 0.001, and *** to p values < 0.001.

Statistics were calculated by GraphPad software 9.0.

## Supplementary Information

**Additional file 1: Fig. S1**. Effect of high glucose treatment on IkBα expression over 60 min. **Figure S2**: DMSO, KT5720 and Inh. XII treatment does not significantly alter proteasome activity and composition under resting conditions. **Figure S3**: Silencing of CaMKIIα expression induces a drop of pRpt6(ser120)/Rpt6 ratio. **Figure 4**: DMSO, KT5720 and Inh. XII treatment does not modulate NF-kB signalling pathway under resting conditions. **Figure S5**: Generation of rMC1 clones expressing the Rpt6_S120A phosphodead mutant showing enhanced proteasome activity. **Figure S6**: Rpt6_S120A clones show constitutively elevate NF-kB activation. **Figure S7**: Determination of the dynamic linear range of actin and Ponceau.

## Data Availability

Original data will be made available upon reasonable request to M.D. Ph. D. Diego Sbardella.
