# Supplementary material for: A novel and atypical NF-KB pro-inflammatory program regulated by a CamKII-proteasome axis is involved in the early activation of Muller glia by high glucose
Source: Cell Biosci. 2022 Jul 16;12:108. doi: 10.1186/s13578-022-00839-x (PMC9287993; doi:10.1186/s13578-022-00839-x)
Supplement: Supplementary file 1 — Additional file 1: Fig. S1. Effect of high glucose treatment on IkBα expression over 60 min. Figure S2: DMSO, KT5720 and Inh. XII treatment does not significantly alter proteasome activity and composition under resting conditions. Figure S3: Silencing of CaMKIIα expression induces a drop of pRpt6(ser120)/Rpt6 ratio. Figure 4: DMSO, KT5720 and Inh. XII treatment does not modulate NF-kB signalling pathway under resting conditions. Figure S5: Generation of rMC1 clones expressing the Rpt6_S120A phosphodead mutant showing enhanced proteasome activity. Figure S6: Rpt6_S120A clones show constitutively elevate NF-kB activation. Figure S7: Determination of the dynamic linear range of actin and Ponceau. [file 13578_2022_839_MOESM1_ESM.docx]

**Additional file 1**


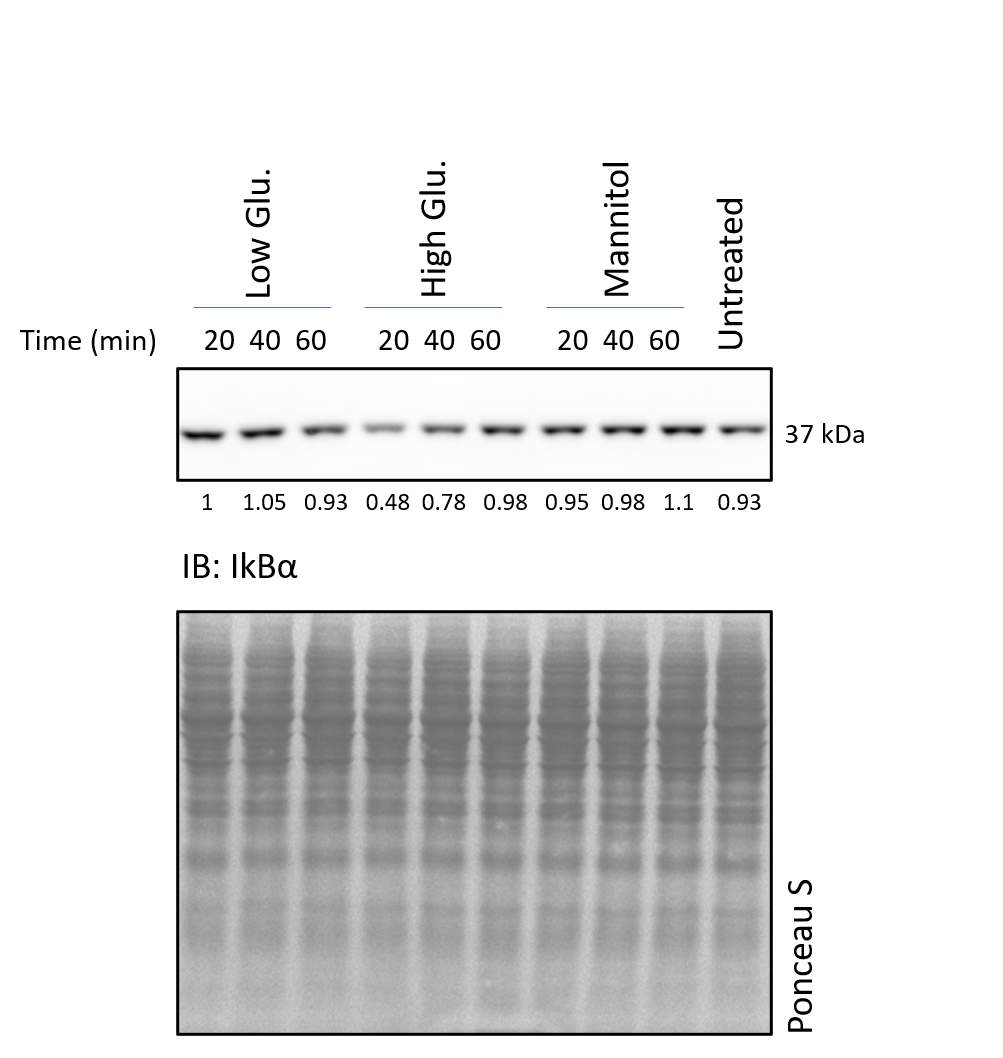


**Fig. S1**: **Effect of high glucose treatment on IkBα expression over 60 min**. Representative immunoblot showing the IkBα pattern 20-40-60 minutes after exposure to low glucose, high glucose and mannitol. Untreated cells were run in parallel. Degradation of IkBα was observed only in the presence of high glucose at 20 min of stimulation. The relative ratio of raw intensity values is reported under each band of the immunoblot.


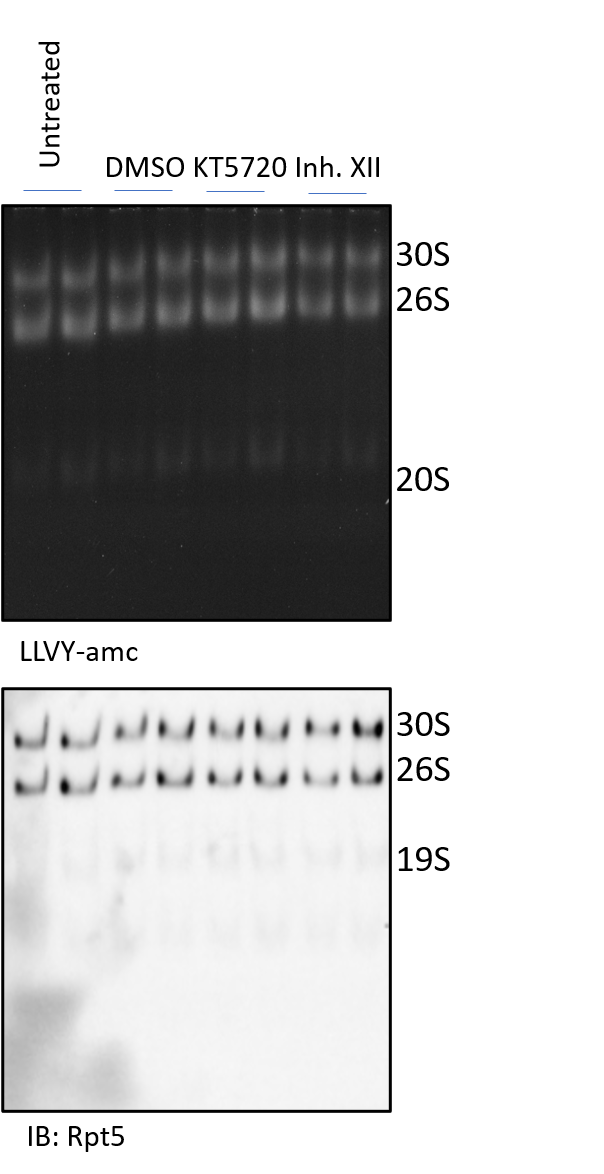


**Figure S2: DMSO, KT5720 and Inh. XII treatment does not significantly alter proteasome activity and composition under resting conditions**. Native gel assay of rMC1 cells grown in low glucose medium and treated with 10 µM Inh. XII and KT5720 or DMSO for 2h, together with untreated cells was performed. Samples were run in duplicate. Proteasome activity was probed in situ with 75 µM LLVY-amc (upper panel) and proteasome particles identified by probing the filter with the anti-Rpt5 antibody (lower panel). No effects were documented on proteasome activity and structural composition.


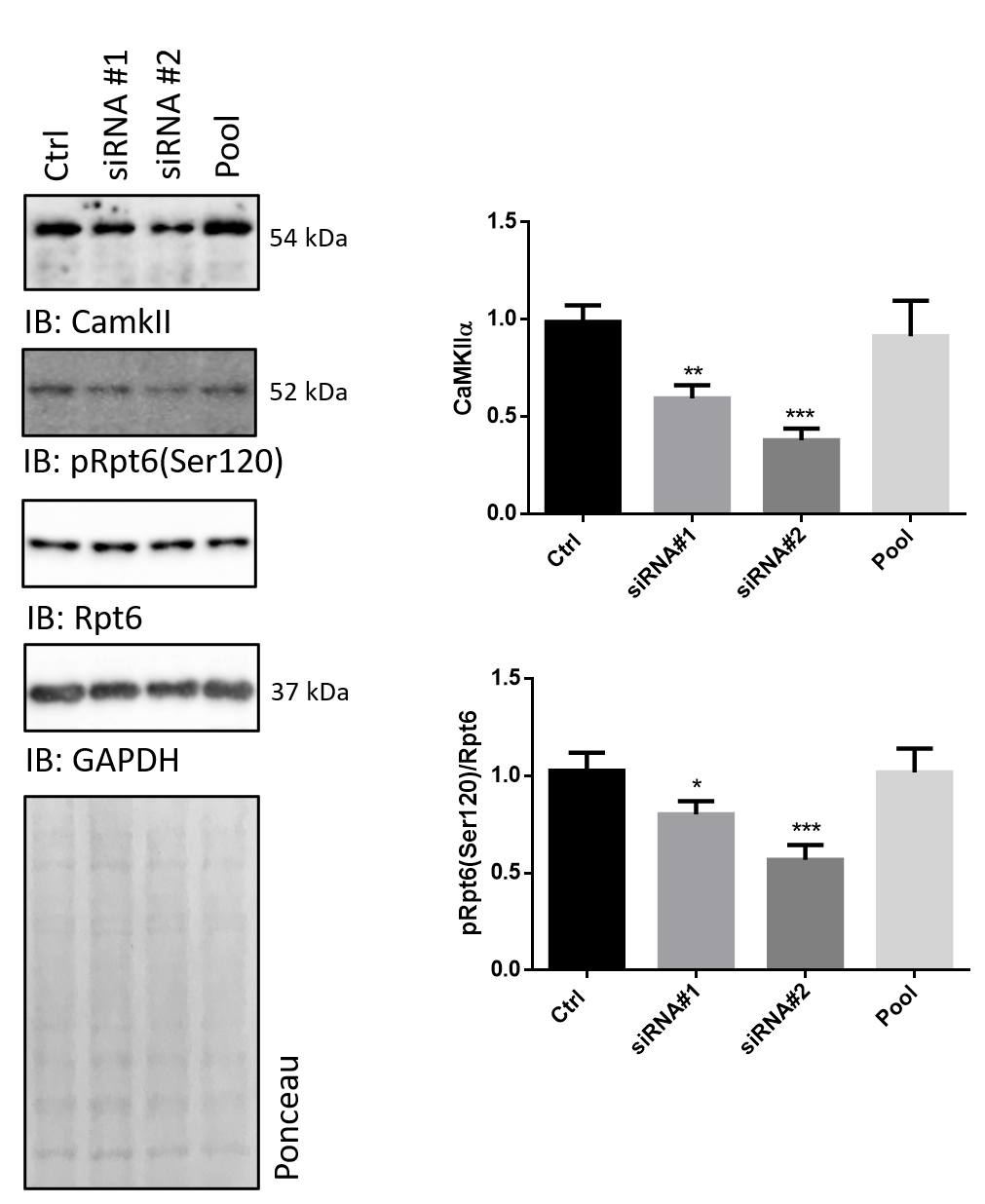


**Figure S3: Silencing of CaMKIIα expression induces a drop of pRpt6(ser120)/Rpt6 ratio.**

rMC1 were cultivated in 5 mmol/L glucose (Low Glu.) and silenced for CaMKIIα expression by delivery of two independent 27-mer siRNA (#1-#2). As internal control, cells were either untreated (Ctrl) or challenged with a non-targeting pool of siRNA (Pool). Whole cell lysates were harvested after 72h from siRNA delivery and assayed for CaMKIIα, pRpt6(ser120) and unphosphorylated Rpt6 content by Wb. With respect to untreated cells or cells challenged with the non-targeting pool, delivery of siRNA#1 and #2 induced a robust drop of CaMKIIα and of pRpt6(ser120) immunostaining. Basal level of unphosphorylated Rpt6 were unaffected by treatment. Thus, the pRpt6(Ser120)/Rpt6 ratio was significantly reduced in CamKIIα-silenced cells with respect to both control and Pool-treated cells. Histograms report the relative abundance of the protein. A nominal value of 1 was assigned to Control cells. Data are presented as mean ±SD (n=4). A representative blot of 2 independent experiments is shown. One-way ANOVA followed by Tukey’s post-hoc test. *p<0.05; **p<0.001; ***p<0.0001;


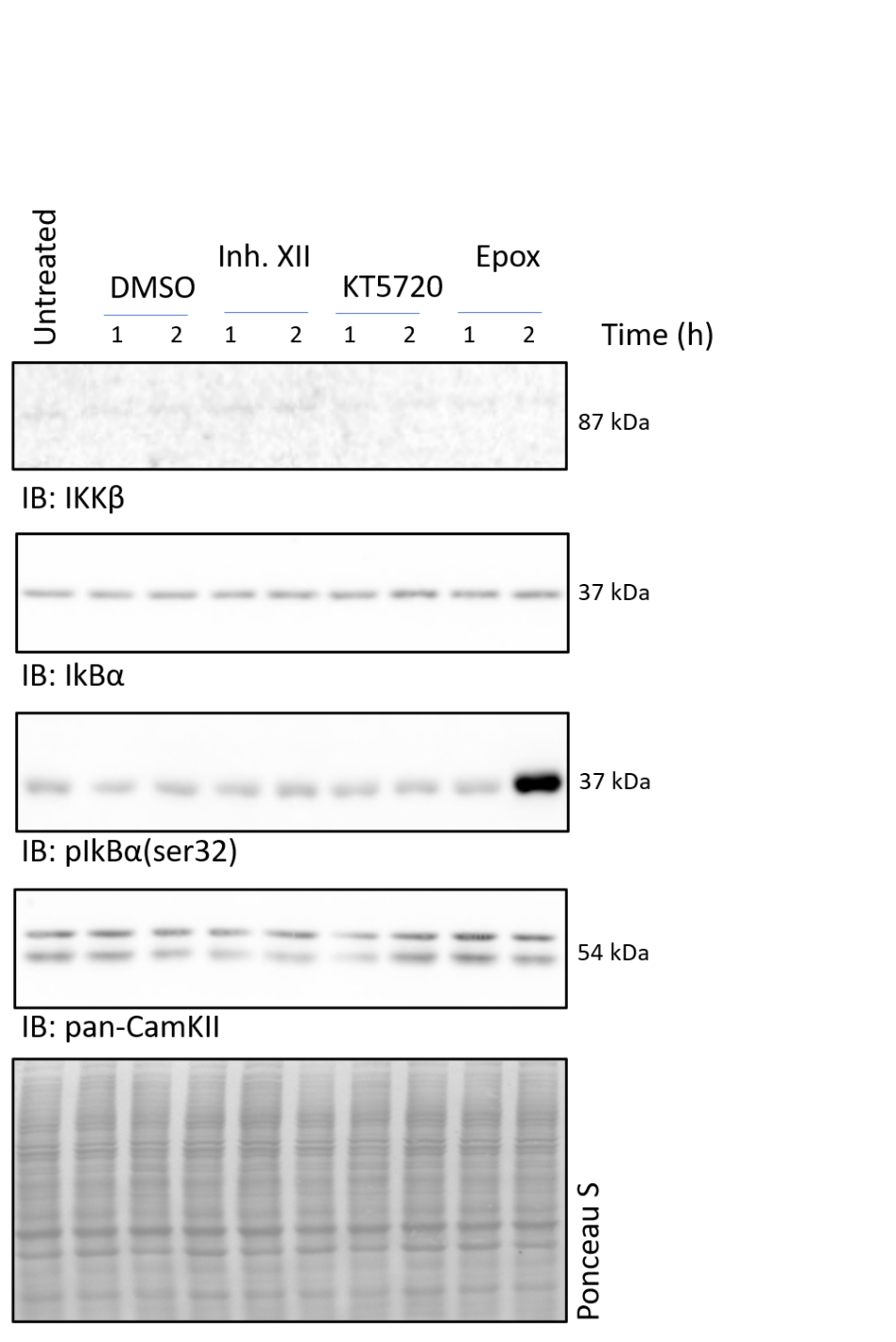


**Figure 4: DMSO, KT5720 and Inh. XII treatment does not modulate NF-kB signalling pathway under resting conditions**. rMC1 cells cultivated in low glucose DMEM were treated with 10 µM Inh. XII or KT5720 or 500 nM epoxomicin, or the equivalent dilution of DMSO, for 1-2h. Thereafter, whole cell lysates were harvested and analysed by Wb. Filters were probed with antibodies against: IKKβ, pIKKβ (not shown because undetectable), IkBα, pIkBα(ser32) and pan-CamKII. No modulation of the protein was induced by these compounds, with the exception of pIkBα(ser32) which turned out to be significantly increased by epoxomicin after 2h of treatment.

A


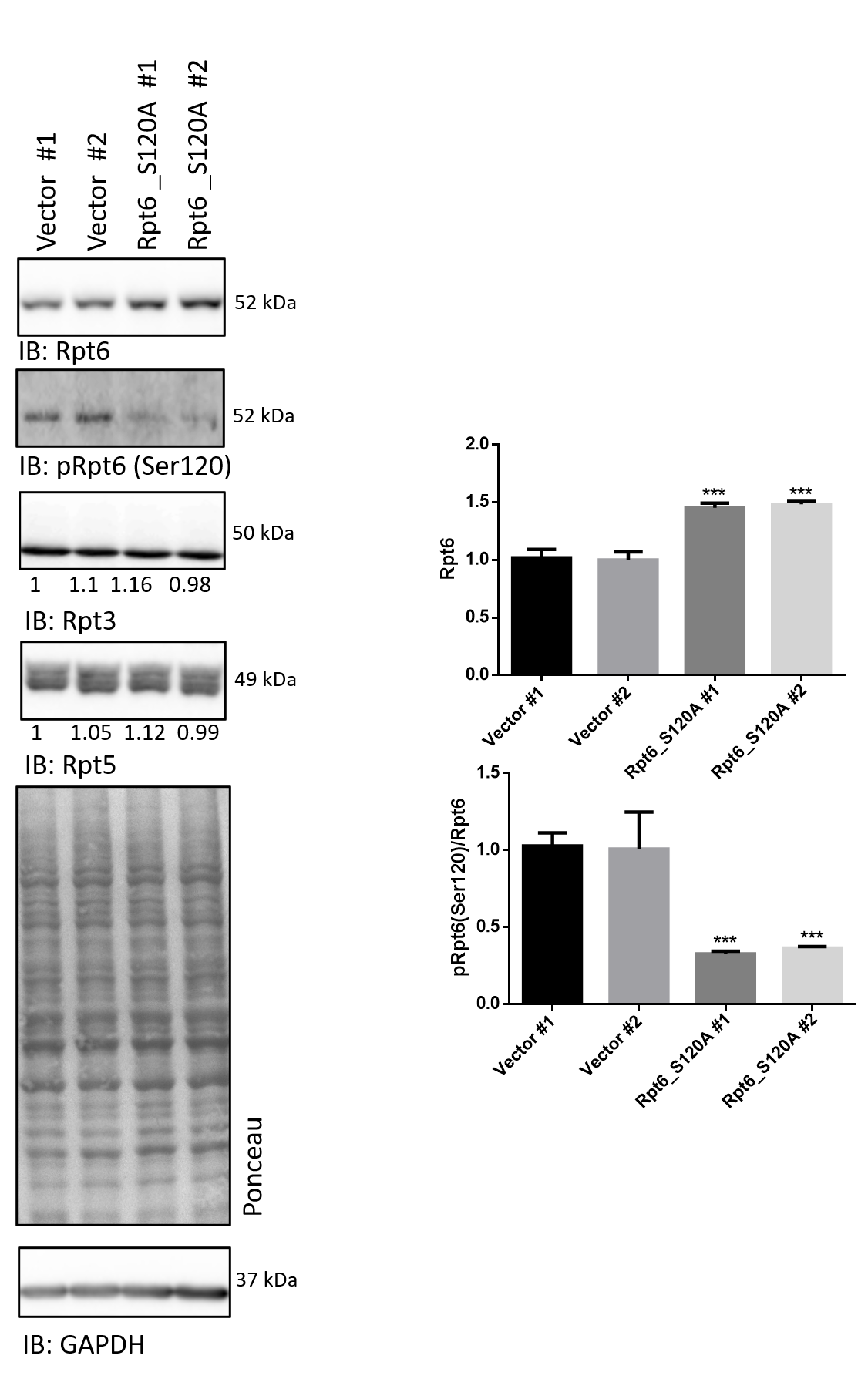


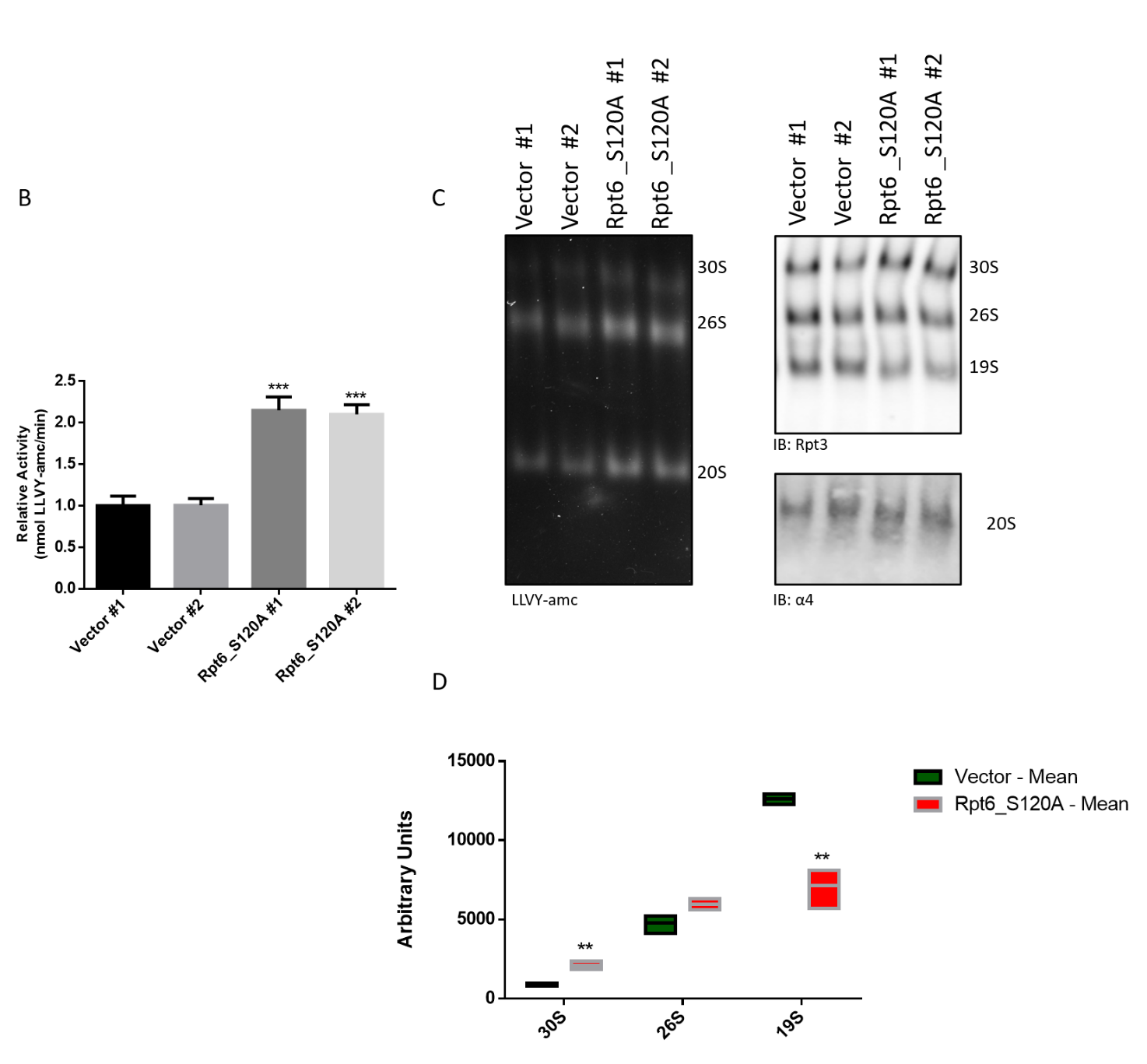


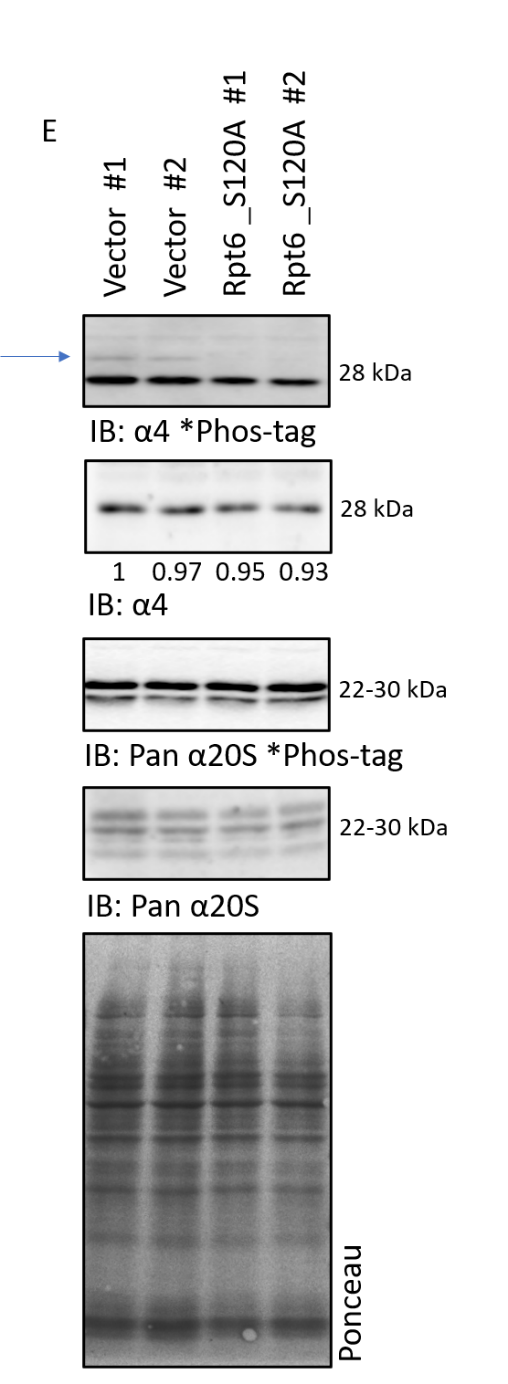


**Figure S5:** **Generation of rMC1 clones expressing the Rpt6_S120A phosphodead mutant showing enhanced proteasome activity.** (A) increased Rpt6 immunostaining along with decreased pRpt6(ser120)/Rpt6 ratio of rMC1 transfected with the plasmid encoding for the Rpt6 phospho-dead mutant. Vector and Rpt6_S120A clones were assayed for Rpt6 and pRpt6(ser120) content by Wb. In parallel, Rpt3, Rpt5 and GAPDH, as loading control, were further assayed. Rpt6_S120A cells display a nearly 50% increase of total Rpt6 with respect to Vector clones. Immunostaining of the protein by a phospho-specific antibody revealed a marked decrease of pRpt6(ser120) immunostaining in Rpt6_S120A cells. Histograms refer to densitometry of bands immunoreactive for Rpt6 or for the pRpt6(ser1320)/Rpt6 ratio (n=3). A representative blot of 5 independent experiments is shown. One-way ANOVA followed by Tukey’s post-hoc test. ***p<0.001; (B) Crude cell extracts were isolated from Rpt6_S120A and Vector clones and assayed for bulk chymotrypsin-like activity as indicated in Fig. 4C. Data are presented as mean ±SD (n=3). A representative experiment is shown. One-way ANOVA followed by Tukey’s post-hoc test (n=3): ***p<0.001; (C) the same crude cell extracts were analysed by native-gel electrophoresis and the particles probed in situ for proteasome activity was probed in situ with 75 µM LLVY-amc (left panel) and proteasome particles identify by probing the filter with anti-Rpt5 and -α4 (20S) antibodies (right panels); (D) Intensities of 30S, 26S and free 19S in the Wb analysis shown in panel B was determined. Raw densitometric values are reported to highlight the relative abundance of the three particles. Data are presented as mean ±SD (n=3). One-way ANOVA followed by Tukey’s post-hoc test: **p<0.01; (E) whole cell lysates were prepared from Rpt6_S120A and Vector clones and analysed by phos-Tag and Wb. Filters were stained with an anti-α4 antibody and with an anti-pan-α20S antibody which targets all 20S α-subunits with the exception of α4.


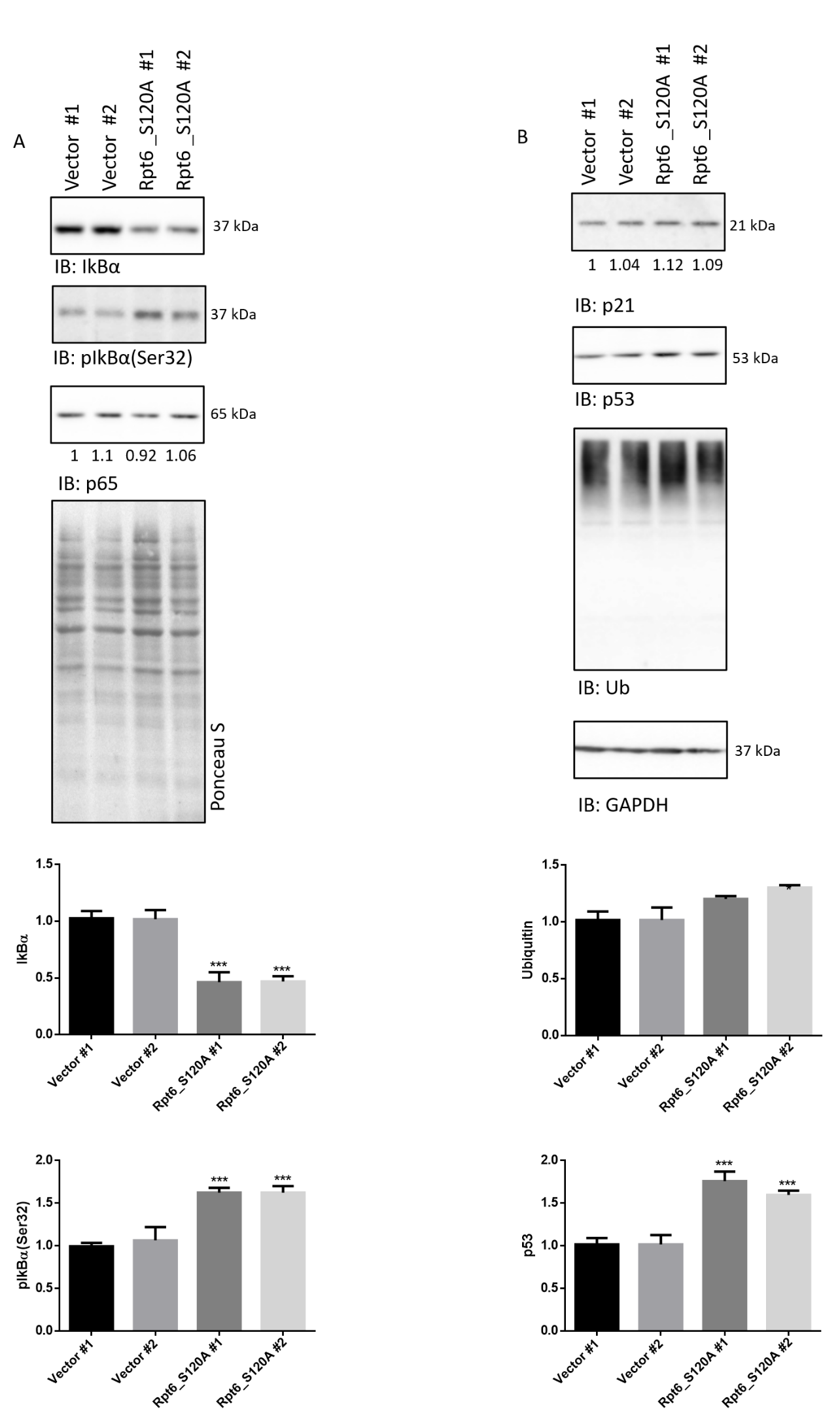


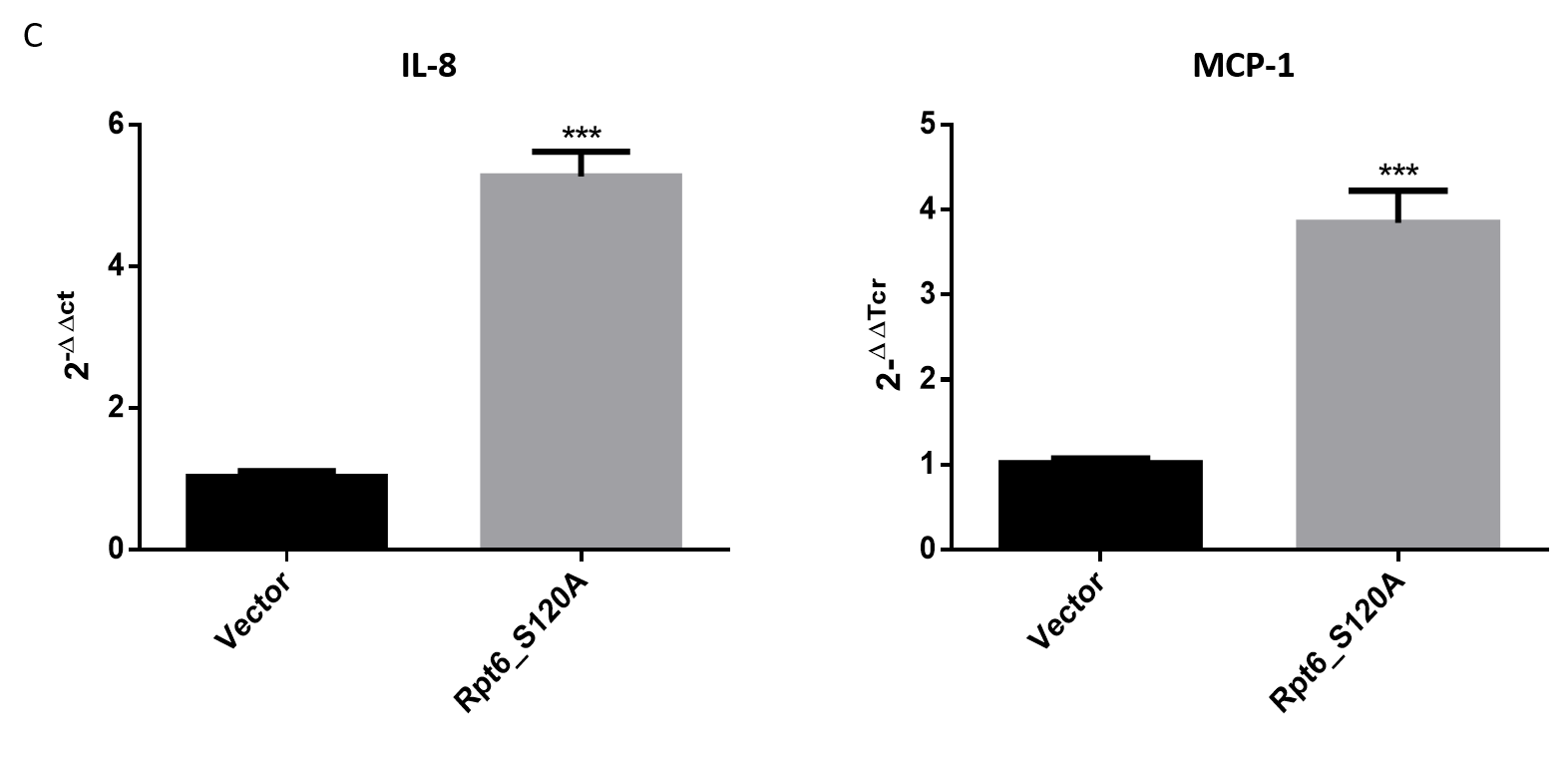


**Figure S6: Rpt6_S120A clones show constitutively elevate NF-kB activation.**

(A) Whole cell lysates were prepared from Rpt6_S120A and Vector clones and analysed by Wb. Filters were stained with the anti-IkBα, -pIkBα(ser32) and -p65 antibodies; total proteins stained by Ponceau S were used as loading control; (B) the same lysates were further probed with antibodies raised against the natural proteasome substrates, p53, p21 and poly-ubiquitinylated proteins. GAPDH was used as loading control. Histograms in (A) and (B) report the densitometry of immunoreactive bands of clones. Data are presented as mean ±SD (n=3); a representative blot of three independent replicates is shown; statistics refers to differences between Rpt6_S120A vs Vector clones. one-way ANOVA followed by Tukey’s post-hoc test: ***p<0.001; in (A) and (B) histograms shown are limited to those for which a statistical significance was obtained; (C) transcripts of IL-8 and MCP1 together with IL-1β (data not shown) of Rpt6_S120A and Vector clones were assayed by RT-PCR. β-actin was used as internal control. Data are presented as mean ±SD (n=3). One-way ANOVA followed by Tukey’s post-hoc test (n=3): ***p<0.001.


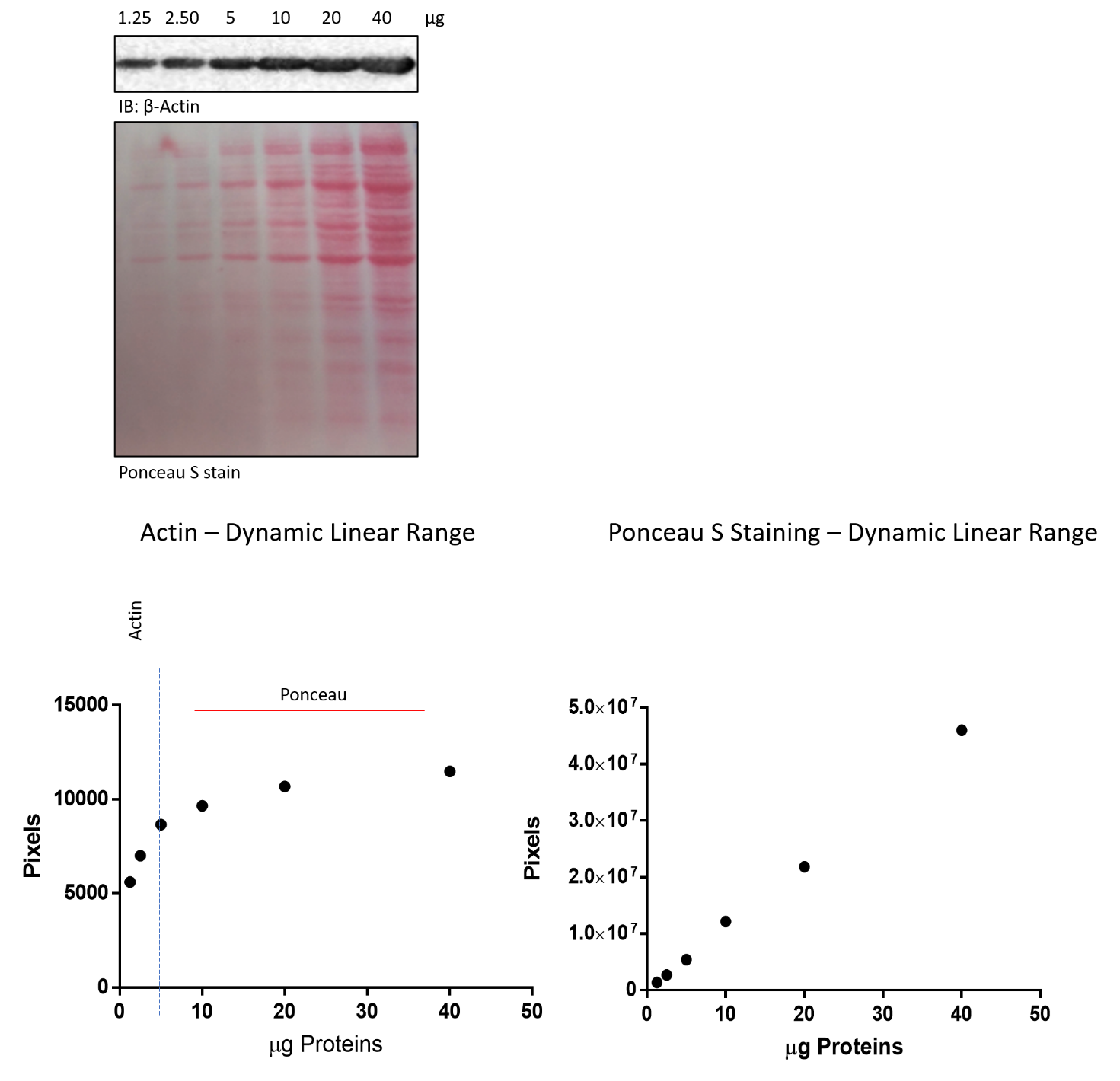


**Figure S7**: **Determination of the dynamic linear range of actin and Ponceau.** Actin, but also GAPDH (not shown) detection is off the linear range at 7.5 µg of proteins loaded per well (as indicated by the appearance of ghost bands over this threshold). Ponceau S, though less sensitive, displays a linear range up to 40 µg of proteins. The choice of using actin or Ponceau was based on the µg needed to get a valid immunoblot for any given target protein.
